# Multi-Omics Characterization of E3 Regulatory Patterns in Different Cancer Types

**DOI:** 10.3390/ijms25147639

**Published:** 2024-07-11

**Authors:** Zhongyan Li, Jingting Wan, Shangfu Li, Yun Tang, Yang-Chi-Dung Lin, Jie Ni, Xiaoxuan Cai, Jinhan Yu, Hsien-Da Huang, Tzong-Yi Lee

**Affiliations:** 1 Warshel Institute for Computational Biology, School of Medicine, The Chinese University of Hong Kong, Shenzhen 518172, China; 219059027@link.cuhk.edu.cn (Z.L.); 118010284@link.cuhk.edu.cn (J.W.);; 2 Department of Biological Science and Technology, National Yang Ming Chiao Tung University, No. 75, Boai Street, Hsinchu 300, Taiwan

**Keywords:** ubiquitination, proteomics, multi-omics analysis, cancer, E3 ubiquitin ligases

## Abstract

Ubiquitination, a post-translational modification, refers to the covalent attachment of ubiquitin molecules to substrates. This modification plays a critical role in diverse cellular processes such as protein degradation. The specificity of ubiquitination for substrates is regulated by E3 ubiquitin ligases. Dysregulation of ubiquitination has been associated with numerous diseases, including cancers. In our study, we first investigated the protein expression patterns of E3 ligases across 12 cancer types. Our findings indicated that E3 ligases tend to be up-regulated and exhibit reduced tissue specificity in tumors. Moreover, the correlation of protein expression between E3 ligases and substrates demonstrated significant changes in cancers, suggesting that E3-substrate specificity alters in tumors compared to normal tissues. By integrating transcriptome, proteome, and ubiquitylome data, we further characterized the E3-substrate regulatory patterns in lung squamous cell carcinoma. Our analysis revealed that the upregulation of the SKP2 E3 ligase leads to excessive degradation of BRCA2, potentially promoting tumor cell proliferation and metastasis. Furthermore, the upregulation of E3 ubiquitin–protein ligase TRIM33 was identified as a biomarker associated with a favorable prognosis by inhibiting the cell cycle. This work exemplifies how leveraging multi-omics data to analyze E3 ligases across various cancers can unveil prognosis biomarkers and facilitate the identification of potential drug targets for cancer therapy.

## 1. Introduction

Ubiquitination is a post-translational modification (PTM) in which a small protein called ubiquitin is covalently attached to target proteins. This modification plays a crucial role in various cellular processes, including protein degradation, signal transduction, DNA repair, and cell cycle regulation. Ubiquitination is carried out by a cascade of enzymatic reactions involving three main types of enzymes: ubiquitin-activating enzymes (E1), ubiquitin-conjugating enzymes (E2), and ubiquitin ligases (E3). E3 ligases determine the specificity of ubiquitination by recognizing target proteins and facilitating the transfer of ubiquitin from E2 to the target protein. The opposite enzyme of E3 is deubiquitinases (DUBs). Deubiquitinases are a group of enzymes that remove ubiquitin moieties from proteins, counteracting the effects of ubiquitin ligases [1]. DUBs, E1, E2, and E3 collectively control the turnover of ubiquitin chains on target proteins. The topology of ubiquitin chains sends different signals for protein degradation and signal transduction. For instance, the K48-linked chain is the most prevalent type of ubiquitin chain, typically leading to the degradation of ubiquitinated proteins. In contrast to K48-linked chains, K63-linked chains do not typically target proteins for degradation. Instead, they play key roles in cellular processes such as DNA repair, endocytosis, and immune signaling [2]. Dysregulation of ubiquitination has been associated with numerous diseases, including cancer, neurodegenerative disorders, and immune system dysfunction [3,4].

E3 ligases are responsible for the specificity of substrates, thereby dictating the fate and function of the modified proteins. Aberrant regulation of E3 ligases has emerged as a significant factor in cancer pathogenesis. For instance, the E3 ligase MDM2 is known to target the tumor suppressor protein p53 for ubiquitination and subsequent degradation, leading to the loss of p53-mediated cell cycle arrest and apoptosis. Overexpression of MDM2 is frequently observed in various cancers, resulting in the suppression of p53 activity and promoting tumor growth [5]. Research on E3 ligases is of great importance also due to their potential as therapeutic targets for drug development. Understanding the regulatory mechanisms of ubiquitination and the roles of E3 ligases in disease processes can provide valuable insights for developing novel therapeutic interventions. One promising approach in drug development is the use of PROTACs (Proteolysis Targeting Chimeras), which utilize the ubiquitin–proteasome system to selectively degrade disease-causing proteins. PROTACs work by recruiting E3 ligases to target proteins of interest, leading to their ubiquitination and subsequent degradation. This strategy has gained attention as a potential therapeutic avenue for various diseases, including cancer and neurodegenerative disorders [6].

Ubiquitination leading to the degradation of substrates regulates protein abundance independently of mRNA expression level. This means that when a protein with a specific transcription level has a higher level of ubiquitination, it is likely to be sent to the proteasome for degradation, thus having a lower protein level. It has been reported that for the correlation between mRNA and protein log2-transformed fold changes between human early-onset gastric cancer tissues and adjacent normal tissues, only 34.3% of genes showed a significantly positive correlation [7]. Therefore, proteomics plays an irreplaceable role in ubiquitination research. Through proteomic analysis, researchers can uncover global changes in protein expression patterns under various physiological and pathological conditions, providing a comprehensive view of the ubiquitin–proteasome system and its functional implications. By integrating proteomic data with other omics datasets, such as transcriptome and interactome, a more comprehensive understanding of the regulatory mechanisms underlying protein ubiquitination can be achieved. Previous computational analyses of E3 ligases and DUBs in cancers primarily focused on mutations in the cancer genome. Some of these analyses have been complemented by integrating transcriptomic data, as well as limited-scale proteomic data such as those generated by Reverse Phase Protein Array (RPPA). RPPA is a technique used to measure the relative levels of specific proteins or PTM. However, RPPA is typically designed to measure a predefined set of proteins or PTM. The coverage of proteins or modifications is limited by the availability of specific antibodies. This means that RPPA data may not capture the entire proteome or encompass all relevant proteins involved in a specific biological process or disease. A representative study on E3 and DUBs in different cancer types was proposed by Collin Tokheim et al. [8]. This article presented a systematic characterization of mutations that affect protein degradation in human cancers. The authors employed comprehensive genomic and transcriptomic analyses to identify mutations in cancer-related genes that potentially disrupt protein degradation pathways. They investigated the functional consequences of these mutations and their impact on protein stability and turnover. However, the protein expression data they used were generated by RPPA, where the number of proteins covered in each cancer type is just around two hundred. This quantity is far from the approximately 20,000 proteins identified by mass spectrometry.

Therefore, in our study, we collected global proteomic data from 12 different cancer populations. We investigated the expression patterns of E3 ligases and DUBs across 12 cancers. By integrating transcriptome, proteome, and ubiquitinome, we constructed a comprehensive landscape of E3-substrate regulatory patterns in lung squamous cell carcinoma (LSCC), which uncovers dysregulated E3 ligases and their specific substrates, and identified the potential prognostic biomarker and the molecular mechanisms underlying their involvement in cancer progression. This information can provide valuable insights into potential therapeutic targets for cancer treatment.

## 2. Results

### 2.1. Assembly of Proteomic Data from Diverse Cancer Cohorts

**Figure 1:**
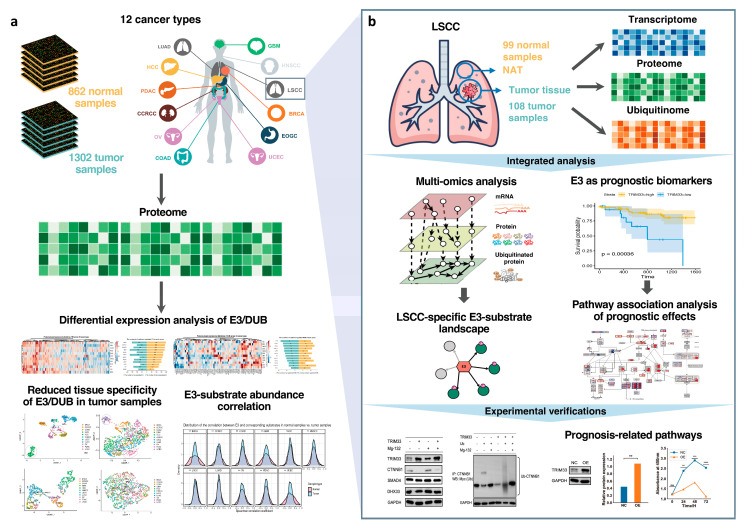
Schematic diagram illustrating the systematic analysis based on multi-omics data from various cancers. (**a**) The investigation based on proteomics data for 12 cancers. (**b**) The systematic analysis based on multi-omics data for LSCC. * *p*-value < 0.05; ** *p*-value < 0.01; *** *p*-value < 0.001; **** *p*-value < 0.0001; ns, not significant.

The study curated proteomic data sets of 12 cancer types from the National Cancer Institute’s Proteomic Data Commons (PDC) [9] (Figure 1a and Table 1, see Supplementary Materials and Methods). Among these, there are ten cancer cohorts from the Clinical Proteomic Tumor Analysis Consortium program, and the other two cancer cohorts are from the International Cancer Proteogenome Consortium. Tumor samples and paired normal adjacent tissue (NAT) were collected in ten cancer cohorts, while the normal samples of ovarian serous cystadenocarcinoma (OV) and glioblastoma (GBM) cohorts were not paired with tumor samples (Table 1). Ubiquitinome was only examined for Lung Squamous Cell Carcinoma (LSCC) cohorts. All the proteomic data sets were individually subjected to differential expression analysis (Figure S1). The proteomic investigation of E3 expression in the various cancers, including differential expression analysis, clustering, and correlations between E3 and substrates, revealed E3 regulatory patterns in tumorigenesis. Furthermore, by combining transcriptome and ubiquitinome, the multi-omics analysis for LSCC elucidated LSCC-specific E3-substrate landscape, the E3 prognostic biomarker and related signaling pathways, with experimental validation (Figure 1b).

We collected the known E3 ubiquitin ligases (E3) and deubiquitinases (DUBs). The complete lists of 509 human E3 and 100 DUBs were compiled through querying databases and manual literature review (see Tables S1 and S2, Supplementary Materials and Methods). We then counted the identified E3 and DUBs in proteomic data sets. The numbers of identified E3 and DUBs varied among different types of cancer, with GBM showing approximately twice as many E3 and DUBs compared with COAD. The numbers of E3 and DUBs show positive correlations across different types of cancer, with cohorts having a higher number of E3 also exhibiting a higher number of DUBs (Figure S2)

### 2.2. Proteome-Based Expressional Alterations of E3 Ligases and Deubiquitinases between Tumor and Normal Samples

**Figure 2:**
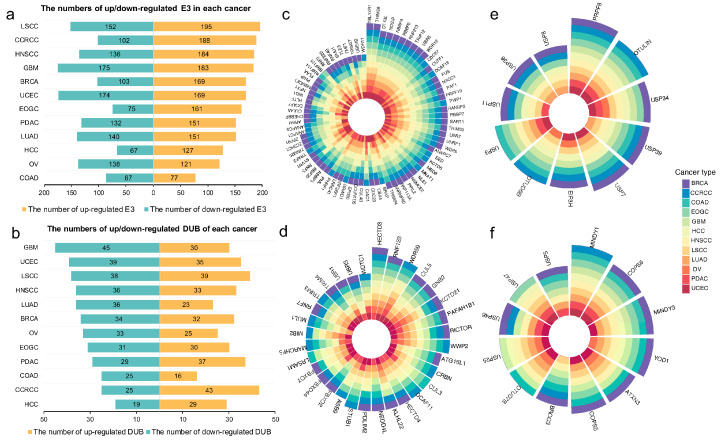

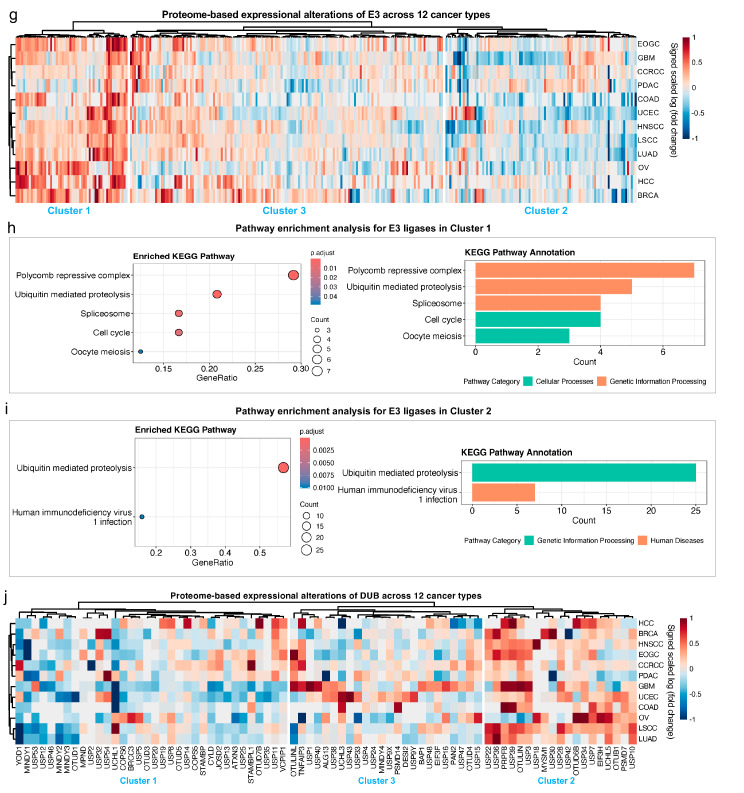
The landscape of regulatory patterns of E3 and DUBs of tumor compared with normal samples. (**a**). The number of up/down-regulated E3 in 12 cancer types (up-regulation: log2FC > 0; down-regulation: log2FC < 0). (**b**). The number of up/down-regulated DUB in 12 cancer types (up-regulation: log2FC > 0; down-regulation: log2FC < 0). (**c**). Significantly up-regulated E3 in most cancer types (adjusted *p*-value < 0.05, log2FC > 0). (**d**). Significantly down-regulated E3 in most cancer types (adjusted *p*-value < 0.05, log2FC < 0). (**e**). Significantly up-regulated DUBs in most cancer types (adjusted *p*-value < 0.05, log2FC > 0). (**f**). Significantly down-regulated DUBs in most cancer types (adjusted *p*-value < 0.05, log2FC < 0). (**g**). Heatmap of proteome-based expressional alterations of E3. The color of each cell depicts the signed scaled log2FC between tumor and normal samples, and the cells in gray represent missing values. (**h**). Dot plot of KEGG pathway enrichment analysis for the E3 belonging to Cluster 1 in (**g**) and pathway category annotation. (**i**) Dot plot of KEGG pathway enrichment analysis for the E3 belonging to Cluster 2 in (**g**) and pathway category annotation. (**j**) Heatmap of proteome-based expressional alterations of DUBs.

We illustrated the numbers of up-regulated and down-regulated E3 and DUBs, respectively, in 12 cancer types. Distinct regulatory patterns are observed between E3 and DUBs, while a common regulatory pattern for either E3 or DUBs is present across cancer types (Figure 2a,b). More E3 were up-regulated in most of the cancer types, while there was more down-regulated DUBs than up-regulated DUBs in most cancer types. It is known that the functions of E3 and DUBs are opposite. E3 catalyze the attachment of ubiquitin molecules to substrates; DUBs remove these ubiquitin molecules from substrates. The regulatory patterns of E3 and DUBs in these proteomic data sets indicate that tumor samples have more ubiquitinated proteins. This is consistent with previous studies in LSCC [10,11], showing increasing ubiquitination sites in tumors compared with normal samples. Differentially expressed E3 and DUBs in more than six cancer types were screened (adjusted *p*-value < 0.05, Figure 2c–f); theypossibly play essential roles in tumorigenesis. For example, TBL1XR1, one of the significantly up-regulated E3 across 12 cancer types, has been reported to be associated with the progression and clinical prognosis of various human malignancies, such as human non-small cell lung cancer [12] prostate cancer [13], extranodal lymphoma [14], and gastric cancer [15].

The complete proteome-based expressional alterations of tumor samples compared with normal samples of E3 and DUBs were presented in Figure 2g and j, respectively, and the one combining E3 and DUBs is shown in Figure S3. E3 and DUBs can generally be separated into three clusters, with ‘Cluster 1’ showing upregulation in most cancers, ‘Cluster 2’ exhibiting downregulation across different cancers, and ‘Cluster 3’ displaying diverse regulatory directions in different cancer types (Tables S3 and S4). When integrating E3 and DUBs, we found that E3 show a propensity for upregulation across multiple types of cancer. In contrast, DUBs tend to be down-regulated across various cancer types (Figure S3). Pathway enrichment analysis results indicate that E3 in ‘Cluster 1’, in addition to being associated with ‘Ubiquitin mediated proteolysis’, as expected, are also linked to genetic information processing including ‘Spliceosome’ and ‘Polycomb repressive complex’ pathways, and fundamental cellular processes including ‘Cell cycle’ and ‘Oocyte meiosis’ (Figure 2h, Table S5). It is evident that these functions involve the fundamental distinctions between cancer and normal cells, irrespective of their tissue of origin, ultimately leading to uncontrolled proliferation. The E3 in ‘Cluster 2’, generally downregulated across cancers, are significantly over-represented in the ‘Human immunodeficiency virus 1 infection’ pathway (Figure 2i). This implies that the downregulation of these E3 is associated with the widespread immunosuppression observed in cancers. In contrast, the regulation directions of E3 in ‘Cluster 3’ vary, potentially exerting diverse effects in various cancers, thus resulting in a dispersed range of functions (Figure S4). 

A previous study focusing on ubiquitin-specific-processing proteases (UPSs), the largest subfamily of DUBs, reported that UPSs show a consistent direction of transcriptome-based expressional alterations in tumor compared with normal samples [16]. Interestingly, in our study, when we examined the proteome-base expressional changes and expanded the scope to include all DUBs, most DUBs exhibited distinct regulatory patterns across cancer types. This may enhance or attenuate their deubiquitinating function on substrates, thereby playing disparate roles in different cancers. Due to their limited numbers, generally upregulated DUBs do not significantly enrich in any pathway (adjusted *p*-value > 0.05). Those in Cluster 2 and 3 enrich in ‘RIG-I-like receptor signaling pathway’ which is responsible for detecting viral pathogens and generating innate immune responses, and the ‘Polycomb repressive complex’ related to genetic information processing, respectively (Figure S5, Table S6). The result highlights the indispensability and necessity of proteome-based analyses in unraveling the ubiquitin system in cancer. The analysis provides a systematic view of E3 and DUB expression patterns in cancers.

### 2.3. Reduced Tissue Specificity of E3 Expression Patterns in Tumor Samples Compared With Normal Samples

**Figure 3:**
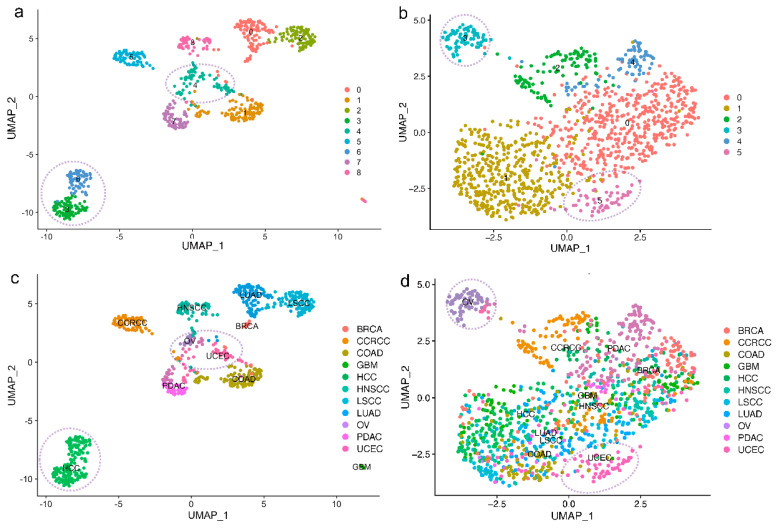

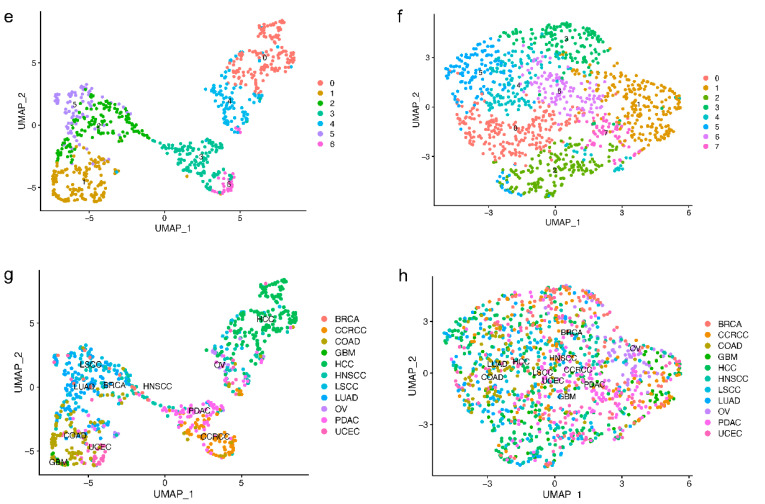
Reduced tissue specificity of E3 expression patterns in tumor samples compared with normal samples. (**a**). Umap of normal samples based on E3 expression colored by cluster identities. (**b**). Umap of tumor samples based on E3 expression colored by cluster identities. (**c**). Umap of normal samples based on E3 expression colored by cancer types. (**d**). Umap of tumor samples based on E3 expression colored by cancer types. (**e**). Umap of normal samples based on DUB expression colored by cluster identities. (**f**). Umap of tumor samples based on DUB expression colored by cluster identities. (**g**). Umap of normal samples based on DUB expression colored by cancer types. (**h**). Umap of tumor samples based on DUB expression colored by cancer types.

To investigate the expression patterns of E3 and DUB in tumor and normal tissues separately, we clustered the tumor samples and normal samples, respectively, across cancer types. The EOGC cohort was excluded from the clustering because the separate proteomics data sets of tumor and normal samples of the cohort were not available. Normalization was employed to remove batch effects, preventing their interference in the subsequent analysis of expression patterns (Figure S6). Based on E3 protein expression, the normal samples from the 11 cancer cohorts were divided into eight clusters, while the tumor samples were only classified into five clusters, with relatively minor distinctions observed between the clusters (Figure 3a,b). This suggests that there is a notable difference of the expression patterns of E3 in normal samples among different cancer types, although only 112 E3 were used in the clustering determination (see Method for details). In addition, the expression patterns of E3 have higher tissue specificity in normal tissues compared with tumor tissues. For tumor samples, those from HCC, LUAD, LSCC, and COAD cohorts were clustered into ‘Cluster 1’ (Figure 3b), while the normal samples from the four cohorts were even divided into five clusters, among which the samples from the HCC cohort were cleaved into two clusters (Figure 3a,c). Tumor samples from BRCA, GBM, and HNSCC cohorts were gathered into ‘Cluster 0’ (Figure 3b,d), while the corresponding normal samples were separated into three clusters (Figure 3a,c). Normal samples from the OV and UCEC cohorts were assigned to ‘Cluster 4’, possibly due to the similar expression patterns of E3 in the normal ovarian and uterine corpus tissues, which are two adjacent reproductive organs (Figure 3a,c). Interestingly, tumor samples of the two cohorts were split apart. This indicates that E3 probably have distinct functional mechanisms in the two types of cancer. In addition, a similar result, that is, the expression patterns of DUBs having higher tissue specificity in normal tissues compared with tumor tissues, was obtained when clustering based on the protein expression of DUBs, indicating that ubiquitin turnover changes dramatically in tumorigenesis (Figure 3e–h).

### 2.4. The Proteome-Based Correlation between E3 and Corresponding Substrates

**Figure 4:**
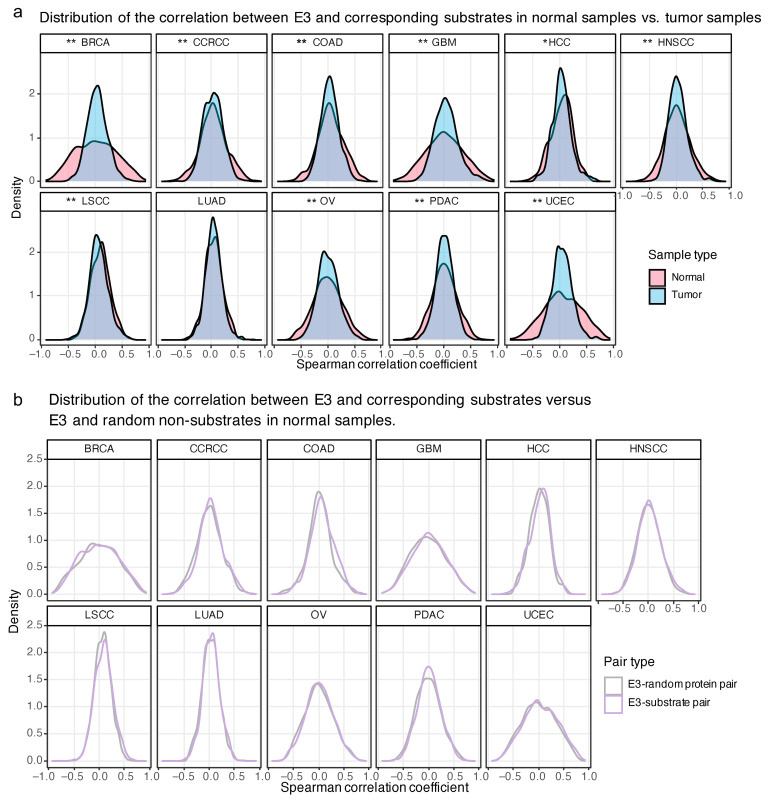

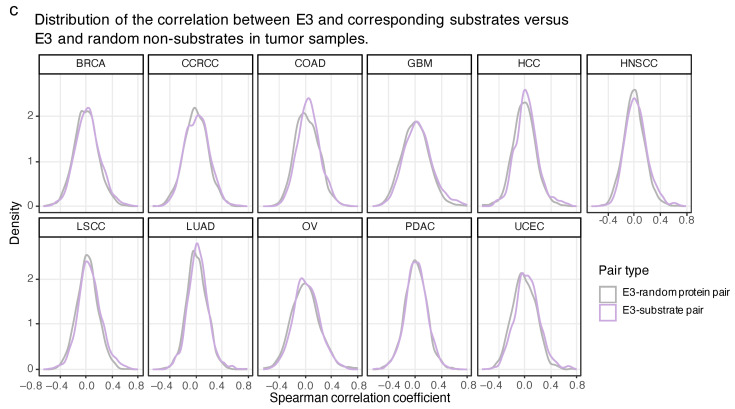
The proteome-based correlation between E3 and corresponding substrates. (**a**). The distribution of Spearman correlation coefficients between E3 and corresponding substrates in normal samples and tumor samples. * significant level of Kolmogorov–Smirnov test: ** adjusted *p*-value < 0.01, * adjusted *p*-value < 0.05. (**b**). The distribution of Spearman correlation coefficients between E3 and corresponding substrates vs. E3 and random non-substrates in normal samples. (**c**). The distribution of Spearman correlation coefficients between E3 and corresponding substrates vs. E3 and random non-substrates in tumor samples.

To dissect the proteome-based relationship between E3 and substrates, we curated the human E3-substrate interactions (ESIs) from multiple sources [17,18,19,20,21,22,23,24,25]. In total, 2922 human experimentally verified ESIs were collected (see Table S7, Supplementary Materials and Methods). It is well known that E3 promote ubiquitin chain formation with different topologies on substrates. Some chain types can lead to proteasomal degradation of substrates, such as those containing K48 linkages, thereby regulating protein turnover and cellular processes. Theoretically, the upregulation of E3 is more likely to lead to the downregulation of their substrates at protein level. To examine the assumption, we calculated the Spearman correlation coefficients between the protein expression of E3 and their substrates in 11 types of cancer. The correlations of tumor samples were compared with those of the normal samples. There was a significant distinction between them in the ten types of cancer (Figure 4a and Table S8). The correlation coefficients of tumor samples were more concentrated in the vicinity of zero, whereas normal samples exhibited a greater proportion of ESIs with positive or negative correlations. This suggests that the known ESIs have undergone alterations in tumor cells compared to normal samples. Furthermore, we tested whether the correlation of ESIs has a greater proportion of negative values compared to E3 and random non-substrate pairs. The result of normal samples is provided in Figure 4b. Notably unexpected, the distribution of correlation between E3–substrate pairs and E3–random protein pairs does not exhibit discernible differences. Moreover, the slightly right shifts of E3–substrate pairs are observed in most of the cancers. The same results of tumor samples are shown in Figure 4c. Interestingly, Piao’s research has also drawn the consistent conclusion that E3 and substrates are not overall negatively correlated at the proteomic level, and the median level of the correlation is near zero. When compared with randomly combined E3 and proteins, ESIs show higher correlations on average [26]. These findings suggest that there is still substantial room for further exploration in E3–substrate regulatory relationships.

### 2.5. Landscape of E3–Substrate Regulatory Pattern in Lung Squamous Cell Carcinoma by Integrating Multi-Omics Data

According to the aforementioned results, it can be inferred that the regulatory relationship between E3 and substrates is complicated. Therefore, integrating ubiquitinome, transcriptome, and proteome is highly necessary. For the 12 cancer cohorts, only the LSCC cohort provided ubiquitinome data. In theory, the abundance of ubiquitination sites on a protein will increase after ubiquitination. However, due to the rapid degradation of ubiquitinated proteins, the actual abundance of ubiquitinated sites needs to be corrected using the expression level of the protein. The original study on the LSCC cohort precisely provided the corrected ubiquitinome data [27]. Therefore, the following analyses were focused on LSCC.

**Figure 5:**
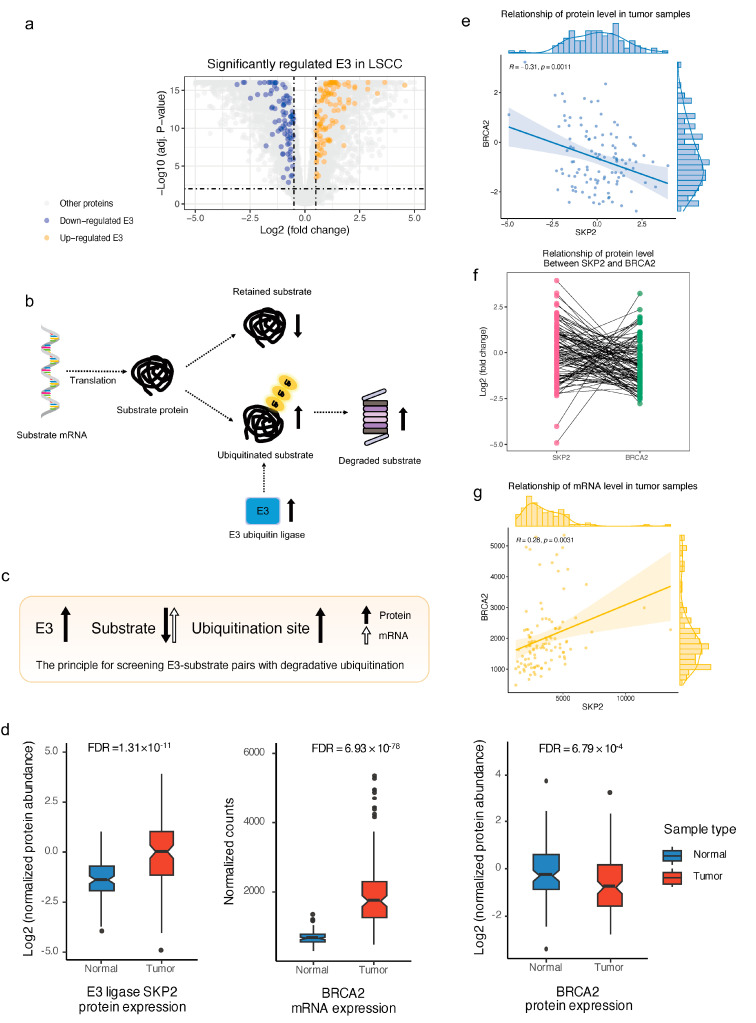
The characterization of E3–substrate regulatory pattern for LSCC. (**a**). The significantly differentially expressed E3 ligases in LSCC. (**b**). The diagram of the impact of degradative ubiquitination on substrates. (**c**). The principle of screening E3–substrate pairs with degradative ubiquitination. (**d**). The protein- and mRNA-based expressional alterations between tumor and normal samples of SKP2 (E3 ligase) and BRCA2 (Substrate). (**e**). The negative correlation of protein level between SKP2 and BRCA2. (**f**). The relationship of protein level between SKP2 and BRCA2. (**g**). The positive correlation of mRNA level between SKP2 and BRCA2.

To identify substrates that undergo excessive ubiquitination and their E3 in LSCC, we screened substrates meeting the following criteria: (A) The substrate has up-regulated ubiquitination sites; (B) At least one known E3 that can target the substrate has up-regulated protein expression in tumor samples. There are 145 significantly up-regulated E3 in the proteomic data set of LSCC (Figure 5a), of which 49 E3 and their 140 substrates met the above requirements. The 140 pairs of ESIs are likely to play important roles in the occurrence and progression of LSCC. Their expressional alterations at mRNA and protein levels compared with normal samples are listed in Supplementary Table S9. It is well known that ubiquitination can be categorized into degradative ubiquitination and non-degradative ubiquitination. It has been reported that approximately 60% of the variation in protein abundance cannot be explained by measuring mRNA independently, which can be attributed to other regulatory processes, including different post-transcriptional regulations, PTM, sequencing depth, and the proteomic approach [28,29]. Excluding technical factors, proteins with up-regulated ubiquitination sites, and low correlation between mRNA and protein level changes are most likely to be over-ubiquitinated and degraded. To narrow down potential substrates undergoing excessive degradation by ubiquitination from the selected ESI and provide more rigorous references, we strictly selected proteins with divergent regulatory directions at the protein and mRNA levels. For example, the protein abundance of substrates with relatively stable or up-regulated mRNA levels can be down-regulated after ubiquitination (Figure 5b). Specifically, the criterion is that the protein abundance of the substrate is down-regulated in LSCC samples, but its mRNA level is not significantly down-regulated, or the protein level of the substrate does not show significant changes, but its mRNA level exhibits a significant increase (Figure 5c). Finally, 18 up-regulated E3 and their substrates primarily regulated by degradative ubiquitination were screened out (Supplementary Table S10). 

One noteworthy E3–substrate pair is S-phase kinase-associated protein 2 (SKP2) and Breast cancer type 2 susceptibility protein (BRCA2). Although the protein abundance severely decreased, the mRNA level of BRCA2 dramatically increased in tumor samples compared with normal samples, which indicates that the downregulation of the BRCA2 protein level was triggered by post-translational modification. In addition, BRCA2 had up-regulated ubiquitination sites, which means that BRCA2 potentially underwent excessive degradation by ubiquitination in LSCC, and this may be caused by the upregulation of SKP2 (Figure 5d). To test this hypothesis, we calculated the correlation between SKP2 and BRCA2. They indeed show a significant negative correlation at the protein level (Figure 5e). This means that in individual LSCC patients, higher expression of SKP2 is associated with a lower protein level of BRCA2 (Figure 5f). Interestingly, this negative correlation was not observed at the mRNA level (Figure 5g), which highlights the irreplaceable role of proteomics in the study of ubiquitination. It has been reported that increased SKP2 expression following the adhesion of prostate cancer cells to basement membranes leads to the degradation of BRCA2 and promotes cell proliferation [30]. This event is a critical phenomenon that occurs at the beginning of the metastatic cascade to local and distant organs. In our study, we identified significantly up-regulated SKP2 and the correspondingly over-degraded BRCA2, which is consistent with the findings of the previous study. This allows for us to formulate a hypothesis that in LSCC, SKP2 induces BRCA2 degradation through excessive ubiquitination, leading to further proliferation of tumor cells and facilitating cancer metastasis. In addition, we observed the preference of E3 regulation for established substrates. For example, E3 ubiquitin–protein ligase TRIM33 (TRIM33) has three reported substrates, namely catenin beta-1 (CTNNB1) [31], ATP-dependent RNA helicase DHX33 (DHX33) [32], and mothers against decapentaplegic homolog 4 (SMAD4) [33]. The ubiquitination sites on DHX33 were not detected by the ubiquitinome, while CTNNB1 and SMAD4 were found to have upregulated ubiquitination sites. According to our multi-omics screening, CTNNB1 was identified as an excessively degraded substrate, while there is no evidence that SMAD4 was ubiquitinated mainly for degradation although TRIM33 is known to cause SMAD4 degradation via its ubiquitin ligase activity [34] (Figure S8, Tables S9 and S10). This indicates that in LSCC, TRIM33 exhibits a preferential regulation of CTNNB1 among the three substrates, which underscores the distinct regulatory relationships between E3 and substrates in specific contexts.

### 2.6. The Upregulation of TRIM33 Is a Biomarker of Favorable Prognosis by Inhibiting Cell Cycle

**Figure 6:**
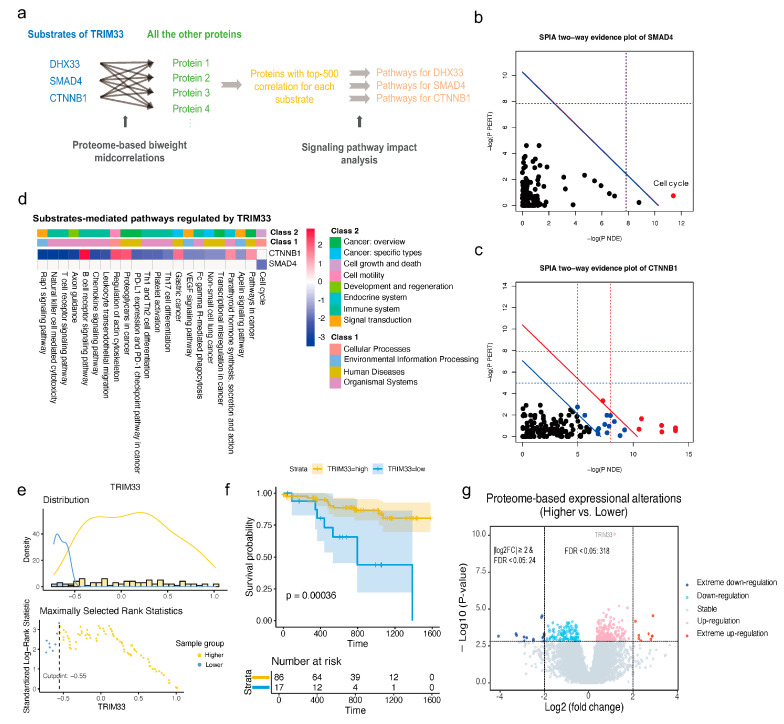
Identification of TRIM33 as a Biomarker for Favorable Prognosis. (**a**). The flow chart of substrate-mediated pathway association analysis. (**b**). SPIA evidence plot for SMAD4-mediated pathways regulated by TRIM33. The horizontal axis represents the negative logarithm of the probability of randomly observing at least a certain number of proteins in the specific pathway. The vertical axis represents the negative logarithm of the probability of randomly observing the total accumulation or more extreme in the specific pathway. Each dot represents a pathway. The dots at the top right of the solid red line are significant pathways after Bonferroni correction of the combined *p*-values using Fisher’s method. The dots at the top right of the solid blue line are significant pathways after an FDR correction of the combined *p*-values. (**c**). SPIA evidence plot for CTNNB1-mediated pathways regulated by TRIM33. (**d**). The substrate-mediated pathways regulated by TRIM33. Blue squares are the inhibited pathways by corresponding substrates, and red squares are the activated pathways. The depth of color squares represents −log10(FDR) of the pathways. (**e**). LSCC samples divided into two groups using the Maximally Selected Rank Statistics method. (**f**). Kaplan–Meier plot based on the two separated groups of samples. (**g**). Protein differential expression analysis based on two divided groups of samples.

Given the crucial roles of E3 in cancer, we investigated whether they have impacts on the prognosis of LSCC patients. We performed survival analysis on 49 significantly up-regulated E3 ligases based on their protein expression levels (Table S11). TRIM33 exhibits a significantly favorable prognostic effect (HR = 0.38, *p*-value = 8.2 × 10^−4^). TRIM33 is a member of the tripartite motif-containing family protein and is involved in many biological processes, such as DNA repair, embryonic development, cell cycle regulation, and immune response [35]. To elucidate the mechanism of the favorable prognostic effect of TRIM33 in LSCC, we performed a substrate-mediated pathway association analysis (Figure 6a). Proteins correlated with the three substrates were used for pathway association analysis. We employed the signaling pathway impact analysis (SPIA) algorithm [36] to assess the impact of these proteins on the pathways (see Supplementary Materials and Methods). SMAD4 was identified to inhibit the ‘Cell cycle’ pathway, which is potentially related to the favorable prognostic effect of TRIM33 in LSCC (Figure 6b,d). There was no pathway associated with DHX33. CTNNB1 was found to be associated with 21 pathways (Figure 6c,d), among which the ‘Non-small cell lung cancer’ pathway was significantly suppressed. It is worth noting that LSCC is precisely one subtype of non-small cell lung cancer. Nevertheless, CTNNB1 simultaneously activated the ‘Pathways in cancer’ and ‘Gastric cancer’ pathways. 

Although we investigated the impact of TRIM33 on downstream biological processes, these effects may not necessarily be the cause of the favorable prognostic effect of TRIM33. Therefore, we further divided LSCC samples into two groups based on the continuous protein expression values of TRIM33. The Maximally Selected Rank Statistics algorithm [37] was utilized to determine the optimal cut-point for TRIM33 expression (Figure 6e). The LSCC samples were divided into two groups: a higher TRIM33 expression group comprising 86 samples and a lower TRIM33 expression group comprising 17 samples. Patients in the higher TRIM33 expression group exhibited significantly better survival outcomes compared to those in the lower group (Figure 6f). The protein expression levels between these two groups were compared, revealing significant differences in the expression of 318 proteins (FDR < 0.05, Figure S9a). Among them, 24 proteins displayed pronounced disparities (FDR < 0.05, log2FC ≥2, Figure 6g). The heatmap presents the distinct expression patterns of the 24 proteins (Figure S9b). The protein regulatory direction of Cluster 1 enriched with samples displaying a lower expression of TRIM33 is nearly opposite to that of Cluster 3, which contains more samples with higher TRIM33 expression. To elucidate the mechanism contributing to the better prognosis of higher TRIM33 expression, all 318 differentially expressed proteins were used for the following pathway association analysis. The ‘Cell cycle’ pathway was eventually identified with 14 proteins enriched in the pathway, and the pathway was significantly inhibited (FDR = 6.5 × 10^−5^). 

**Figure 7:**
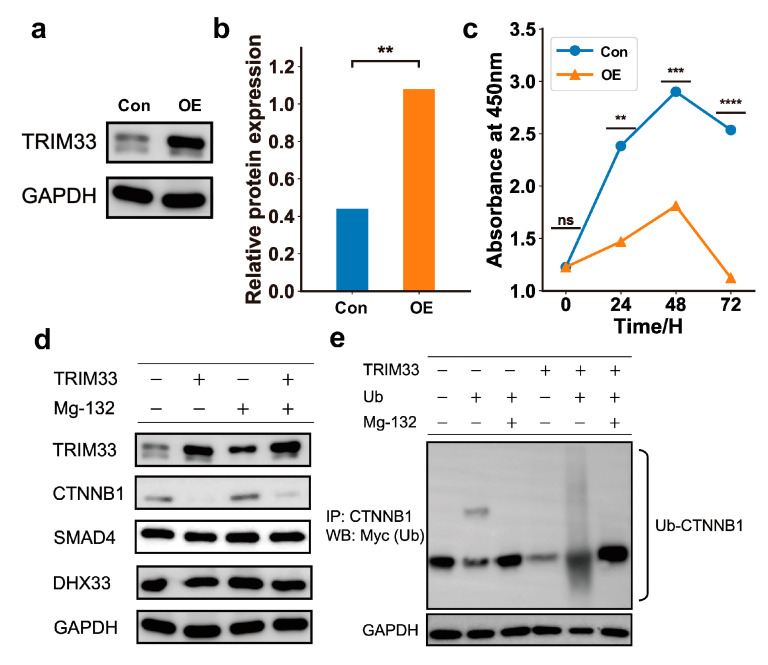
TRIM33 upregulation inhibits cell cycle in the LSCC cohort and ubiquitinates CTNNB1 for degradation. (**a**). NCI-H2170 cells were transfected with TRIM33 expression plasmid. OE, overexpression; Con, control. (**b**). TRIM33 significantly over-expressed in the cells transfected with TRIM33 expression plasmid. ** *p*-value < 0.01. (**c**). Overexpression of TRIM33 can effectively inhibit cell proliferation in H2170 cells. ** *p*-value < 0.01; *** *p*-value < 0.001; **** *p*-value < 0.0001; ns, not significant. (**d**). Western blot the three substrates in cells treated with or without the MG132. Among them, CTNNB1 showed distinct variations between different conditions. (**e**). Western blot showing co-immunoprecipitation of CTNNB1 with Myc-Ub.

To confirm the inhibitory effect of TRIM33 overexpression on the cell cycle, we overexpressed TRIM33 in the H2170 lung squamous cell line (Figure 7a,b) and compared the cell proliferation before and after TRIM33 overexpression (see Supplementary Materials and Methods). The proliferation of H2170 cells was significantly reduced in the TRIM33 overexpression group compared to that observed in the control group at 24 h, 48 h, and 72 h (Figure 7c). To dissect which substrates are strongly ubiquitinated by TRIM33 and the potential consequences of ubiquitination on these substrates, we first compared the stability of the three established substrates of TRIM33 in cells treated with MG132. We observed that TRIM33 overexpression results in a reduction in CTNNB1. Treatment with MG132 can stabilize the CTNNB1 and compensate for the degradation of CTNNB1 caused by overexpression of TRIM33, suggesting that TRIM33 overexpression causes the degradation of CTNNB1 via proteasome in LSCC cells. Comparatively, overexpression of TRIM33 and treatment with MG132 did not have a significant impact on the stability of SMAD4 and DHX33 (Figure 7d). These results support our findings from the multi-omics observation in the previous section. We then examined whether TRIM33 reduces CTNNB1 by ubiquitination. When transfected with the TRIM33 expression plasmid, the ubiquitinated CTNNB1 decreased. Upon the addition of MG132, ubiquitinated CTNNB1 originally subject to degradation was preserved and found to be more abundant compared to the control group (Figure 7e). This result suggests that, in the LSCC cell line, TRIM33 mainly induces the ubiquitination of CTNNB1, leading to its degradation. 

Plus, to identify potential substrates of TRIM33 that may be associated with cell cycle inhibition, we employed the ESI prediction tool, UbiBrowser 2.0 [18], to forecast the substrates of TRIM33. Among the top 20 predicted proteins ranked by the confidence score, four proteins were involved in the ‘Cell cycle’ pathway, namely TP53, MDM2, CCND1, and RB1 (Table S12). The predicted substrates and enriched proteins in pathway analysis on differentially expressed proteins were highlighted in the ‘Cell cycle’ map (Figure S10). The provided map of the ‘Cell cycle’ pathway is expected to provide supporting evidence and clues for future research.

## 3. Discussion

In our investigation into the correlation between E3 and their substrates, we obtained unexpected results. On the one hand, the correlation between E3 and corresponding substrates of tumor samples is significantly different from that of normal samples, which indicates that the ESIs underwent alterations in tumor cells compared to normal samples. It has been reported that the prevalent mutations in cancer potentially caused significant changes in the specificity of protein–protein interaction. Even for mutations at the same site, there was a remarkable divergence in a residue-dependent manner [38]. The typical examples are tumor-suppressor Speckle type POZ protein (SPOP), a substrate-specific adaptor component of a cullin-RING-based BCR E3 complex, and SMAD4. The overlap among mutation-specific interaction partners of SPOP was less than 10%. The G386D and D351H alleles of SMAD4 showed only a 13% fraction of shared interaction partners. Therefore, we speculate that protein–protein interaction reprogramming driven by mutation may exist in tumor tissues of various types of cancer, and this causes the expected correlations between E3 and established substrates are not observed in the tumor samples. 

On the other hand, we found that the correlation between E3 and established substrates at a proteome-wide scale is not more inclined towards a negative correlation compared to the correlation between E3 and random proteins. Interestingly, the prior study also lends support to our findings, indicating that the phenomenon we observed is not merely an incidental result arising from computational errors. We speculate that there are three reasons for this observation. Firstly, we used E3 and randomly selected non-substrates as the control group. However, due to the limited knowledge of ESI, some proteins that have not been identified as substrates may have been categorized as non-substrates, thus affecting the random background distribution. Secondly, although the most abundant ubiquitination signal is K48-linked ubiquitin chains responsible for protein degradation by the proteasome, ubiquitination also functions in a non-degradative manner, such as participating in the regulation of signaling pathways. The ESI with non-proteolytic regulation does not exhibit a negative correlation [39]. Most importantly, although the known ESI have been experimentally validated, they were discovered in diverse experimental backgrounds. The specificity of ESI can also be influenced by factors such as PTM crosstalk of both E3 and substrates, cellular context, and tissue-specific expression patterns [40,41]. For example, other PTMs such as phosphorylation, acetylation, and SUMOylation can alter the conformation of E3 ligases or substrates, create binding sites for interaction partners, or affect the accessibility of target sites for ubiquitination. Consequently, they can fine-tune the specificity and dynamics of ESIs. Therefore, a substantial proportion of substrates may not be negatively regulated by the corresponding E3 in different cancer types. Our results emphasize the importance of considering biological contexts in ESI studies, particularly in the investigation and data compilation of such relationships in different tissues or cells, and physiological states. Presently, the attention given by the existing ESI databases and prediction methods to context-specific ESIs is notably limited, highlighting an area that urgently warrants development.

Due to the complexity of ESIs, the integration of transcriptome, ubiquitinome, and proteome has become imperative. Given the constraint of ubiquitinome, our investigation was confined to LSCC. Through the analyses of multi-omics data, we present an atlas of proteins with up-regulated ubiquitination sites in LSCC, along with their potential E3 ligases. Some of these identified substrates were excessively ubiquitinated for degradation, which were also provided. According to the multi-omics atlas, we speculate that among the three identified substrates of TRIM33, CTNNB1 stands out as a prominent substrate for ubiquitination leading to its degradation in LSCC. This finding is supported by our following experiments. Through survival analysis based on proteomic data, we observed that elevated expression of TRIM33 indicates a longer overall survival for patients. To our knowledge, survival analysis for LSCC based on proteomic data is exceedingly rare, with existing studies predominantly relying on transcriptomic data. In many instances, the transcriptional levels of genes do not align with protein levels [42]. Therefore, we approached the analysis of the impact of E3 ligases on prognosis from a unique perspective. 

TRIM33 has attracted attention due to its undeniable role in various cancers. It was identified as a tumor suppressor in hepatocellular carcinoma [43]. It has also been found that TRIM33 expression is down-regulated in human pancreatic tumors and the inactivation of TRIM33 induces pancreatic precancerous lesions [42]. In renal clear cell carcinoma, TRIM33 regulates tumor progression by inhibiting the TGF-β/Smad pathway and the Wnt signaling pathway [44]. Furthermore, TRIM33 acts as a tumor suppressor by degrading CTNNB1 in human glioblastoma [31]. Here, our study reveals that in LSCC, TRIM33 also functions as a tumor suppressor by inhibiting the cell cycle, thus addressing a gap in elucidating the function of TRIM33 in LSCC.

We additionally proposed the ‘Cell cycle’ map highlighting the key proteins, including the predicted substrates of TRIM33, as well as enriched proteins in pathway analysis on differentially expressed proteins. For example, retinoblastoma-associated protein (RB1), a predicted substrate of TRIM33, is a key tumor suppressor to regulate the G1/S transition of the cell cycle [45]. Unphosphorylated RB1 exerts transcriptional repression on transcription factors E2F1/2/3, thereby inhibiting the expression of genes necessary for cell transcription to enter the S phase. Another predicted substrate of TRIM33 is cellular tumor antigen p53 (TP53). It has been well known that as a transcription factor, p53 can further activate the transcription of genes that inhibit the cell cycle, such as cyclin-dependent kinase inhibitor 1A (CDKN1A) [46]. Serine/threonine–protein kinase ATR (ATR) was significantly up-regulated between the higher and lower TRIM33 expression groups. It phosphorylates TP53, thereby inhibiting DNA replication [47]. TRIM33 appears to be closely associated with the cell cycle from various perspectives. Here, the proposed ‘Cell cycle’ pathway map is expected to provide supporting evidence and clues for further research.

## 4. Conclusions

Our proteome-wide investigation among 12 cancer cohorts shed light on the intricate landscape of E3 and DUB in various cancers. To our knowledge, we are the first to investigate the expression and regulatory patterns of E3 and DUBs at the proteome-wide scale across cancers. Our findings revealed distinct regulatory patterns between E3 and DUBs, with a general trend of upregulation of E3 and downregulation of DUBs in tumor samples. This suggests an increased ubiquitination of proteins in tumor cells. We additionally observed reduced tissue specificity of E3 and DUB expression patterns in tumor samples. The correlation analyses between E3 and substrates unveiled complicated E3–substrate relationships that significantly alter in tumors compared to normal samples. In the investigation of LSCC, we integrated multi-omics data to identify substrates undergoing excessive ubiquitination, such as the CTNNB1 which is preferentially ubiquitinated and degraded by TRIM33, uncovering the LSCC-specific ubiquitination landscape. Notably, the SKP2 and its substrate BRCA2 emerged as a critical regulatory axis with implications for cancer progression and metastasis. We also identified TRIM33 as a significant prognostic biomarker in LSCC, associated with favorable patient outcomes, and its favorable effect is exerted by inhibiting cell cycle. In conclusion, our research provides an atlas for the studies related to the roles of ubiquitination in various cancers. Additionally, we delineate key E3 and substrates pivotal in LSCC, which can serve as a candidate pool for the future development of anti-cancer drugs, given the potential of the ubiquitin–proteasome system in the development of targeted therapy drugs.

## 5. Methods

### 5.1. The Proteome-Based Differential Expression Analysis

Due to variations in data sources and distinct data processing methods employed by each study, we performed individual differential expression analyses for the proteomics data of each cohort. Given the nature of mass spectrometry, it is common to encounter a significant proportion of missing values in the generated data, which affects downstream analyses. Therefore, in our study, proteins with missing expression values in over 50% of the samples were disregarded. For the remaining proteins that still had missing values within the samples, DreamAI (version 0.1.0) was used to fill in the gaps. It is a robust imputation algorithm specifically tailored for proteomics data using crowd learning and consisting of an ensemble of six distinct imputation methods [48]. For the statistical tests of differential expression, the nonparametric Wilcoxon rank-sum test and Wilcoxon signed rank test were employed depending on whether tumor and normal samples were paired. The resulting *p*-values were adjusted using the Benjamini–Hochberg method [49].

### 5.2. Heatmap of Proteome-Based Expressional Alterations

The log2-transformed fold change (log2FC) between tumor and normal samples of E3 and DUB in 12 types of cancer were visualized with a heatmap. The positive values of each column in the heatmap were scaled to the range of 0 to 1; the negative values of each column in the heatmap were scaled to the range of −1 to 0 across 12 cancer types. Two-way hierarchical clustering was performed. The method of clustering was set as ‘ward.D2’ and Euclidean distance was used. The KEGG pathway enrichment analysis for E3 or DUBs of each cluster was conducted by using ‘clusterProfiler’ package (version 4.0) [50].

### 5.3. Cluster Determination of Cohorts across Cancer Types

To investigate the expression patterns of E3 and DUB in tumor and normal samples, respectively, the EOGC cohort, which provides no separate proteomics data sets of tumor and normal samples, was excluded in the following clustering. The remaining proteomics data sets from 11 cancer cohorts exhibited obvious batch effects. We normalized these data sets using the Z-score method for tumor and normal samples, respectively (Figure S6). A large number of E3 and DUBs merely are expressed or identified in particular tissues. To compare the E3 and DUB expression patterns across cancers, we retained E3 and DUBs expressed in all 11 types of cancer. To be specific, there were 114 E3 and 26 DUBs expressed in 1205 tumor samples, and 112 E3 and 25 DUBs expressed in 781 normal samples. Before clustering, we conducted a principal component analysis (PCA) using all the E3 or DUBs expressed in 11 types of cancer as variables, which reduced the dimensionality and discarded the noise. Then, we performed JackStraw analysis for PCA significance (Figure S7). All the significant principal components were employed to determine clusters (*p*-value < 0.05). The cluster determination was conducted using the ‘FindNeighbors’ function, followed by the ‘FindClusters’ function of the ‘Seurat’ package (version 4.1.0) [51]. The resolution of clustering for normal and tumor samples based on E3 expression was 0.5 and 0.6, respectively. The resolution for both normal and tumor samples based on DUB expression was 0.8, considering that only a few DUBs could be used as features.

### 5.4. The Proteome-Based Correlation between E3 and Substrates

We calculated the Spearman correlation coefficient between E3 and their substrates based on protein expression. To determine whether the distributions of correlation coefficients have significant differences between tumor and normal samples, the Kolmogorov–Smirnov test was executed. Furthermore, for each E3–substrate pair expressing in a certain cancer type, we accordingly calculated the correlation between the E3 and a random non-substrate protein. Specifically, we randomly selected a protein that is not the known substrate of the E3 and calculated the correlation between the E3 and the selected protein.

### 5.5. Multi-Omics Analyses for the LSCC Cohort

The E3 meeting the condition of having an adjusted *p*-value of less than 0.01 and an absolute value of log2FC greater than 0.5 were deemed significantly differentially expressed. The RNA-seq count files of the LSCC cohort were downloaded from the National Cancer Institute Genomic Data Commons [52]. The count files were combined into a count matrix following differential expression analysis performed using ‘DEseq2’ package (version 1.34.0) [53]. We computed *p*-values using the Wilcoxon signed-rank test for the different expressions of ubiquitination sites. All the *p*-values were adjusted by using the Benjamini–Hochberg procedure.

Three criteria were used to screen out the substrates potentially undergoing excessive degradation by ubiquitination in LSCC: (a) The substrate has up-regulated ubiquitination sites (log2FC > 0, adjusted *p*-value < 0.05); (b) At least one known E3 that can target the substrate has up-regulated protein expression in tumor samples (log2FC ≥ 0.5, adjusted *p*-value < 0.01); (c) The protein level of the substrate is down-regulated (log2FC < 0, adjusted *p*-value < 0.01), but its mRNA level is not significantly down-regulated, or the protein level of the substrate does not show significant changes, but its mRNA level exhibits a significant increase (log2FC > 0, adjusted *p*-value < 0.01).

### 5.6. TRIM33-Involved Pathway Analysis

The survival analysis was conducted using the Cox proportional hazards model. The up-regulation of TRIM33 was identified as the favorable prognostic factor. Then, we systematically investigated the pathways affected by TRIM33. The downstream effects of TRIM33 were inferred in two steps. It is known that SMAD4, CTNNB1, and DHX33 are substrates of TRIM33. Firstly, for each substrate, we computed the proteome-based bi-weight mid-correlations between the substrate and all the other proteins. Secondly, the proteins were ranked by the absolute values of the correlations, of which the top 500 were used to implement the signaling pathway impact analysis (SPIA, version 2.48.0). Then, to divide the samples into two groups, the Maximally Selected Rank Statistics were used for the evaluation of a cut-point model, which was performed using the ‘maxstat’ package (version 0.7-25) [37]. The 318 differentially expressed proteins between the two groups were used for the signaling pathway impact analysis.

### 5.7. Plasmid Preparation

The plasmid used for overexpressing TRIM33 is pCMV-myc (General Biology, Chuzhou, China), which incorporates a single XbaI restriction enzyme cutting site. The TRIM33 gene was cloned into the plasmids, followed by PCR amplification, restriction enzyme digestion, and ligation. Subsequently, the constructed plasmids carrying the over-expressed TRIM33 gene were introduced into *Escherichia coli* (*E. coli*) through the heat shock transformation method. Finally, plasmid DNA was extracted and purified using Plasmid Mini Preparation Kit (Beyotime, Shanghai, China).

### 5.8. Cell Culture

The H2170 cell line was provided by Guangzhou Cellcook Biotech Co., Ltd. and cultured in Roswell Park Memorial Institute (RPMI) 1640 medium (Gibco, Shanghai, China) with 10% fetal bovine serum (FBS) (Gibco, Shanghai, China) and 1% Penicillin/Streptomycin (p/s) solution (Gibco, Shanghai, China). The H2170 cell was maintained in a controlled environment, cultured at 37 °C, 95% relative humidity, and 5% CO_2_ in a CO_2_ incubator (Yamato Scientific, Shanghai, China).

### 5.9. Transfection

Cells were seeded into 6-well (1 × 10^6^ cells/well) and 96-well (2 × 10^3^ cells/well) plates for further Western blot and CCK-8 assay experiments, respectively. Cells were incubated with RPMI 1640 medium with 10% FBS in 96-well plates. Post 24 h incubation, 100 μL RPMI 1640 medium containing 0.1 ng plasmid DNA and 0.2 μL Lipofectamine™ 2000 Reagent (Invitrogen, Shanghai, China) were used to treat the TRIM33 overexpressed group (OE), while 100 μL RPMI 1640 medium alone acted as control group (NC). Ubiquitin (Ub) plasmids were prepared for transfection (HonorGene, Changsha, China). For 6-well plates, 2 mL RPMI 1640 medium containing 2.5 μg plasmids DNA (2.5 μg TRIM33; 1.25 μg TRIM33 and 1.25 μg Ub; 2.5 μg Ub) and 5 μL Lipofectamine™ 2000 Reagent were used to treat the experimental group.

### 5.10. Cell Counting Kit-8 (CCK-8) Assay

Four hours post transfection, cells were incubated in RPMI 1640 medium with 10% FBS again for 0 h, 24 h, 48 h, and 72 h. A mixture of 10 μL CCK-8 and 90 μL RPMI 1640 medium was added to each column. One hour post incubation, cell proliferation was measured by absorbance at 450 nm (BioTek Gen5, Shanghai, China).

### 5.11. Protein Immuno-Co-Precipitation

Initially, the extracted proteins were prepared and quantified with the BCA method. Specific antibodies, including anti-DHX33 (#ab72451, ABCAM), anti-SMAD4 (#ab230815, ABCAM), and anti-CTNNB1 (#K002207P, Solarbio, Beijing, China), were then introduced into the extract. These antibodies bound to the target protein along with its interacting Ub. Subsequently, Protein A/G-coated beads (Biolab, Guangzhou, China) were added to the mixture, facilitating the sedimentation of antibody-protein complexes. After washing to remove non-specifically bound proteins and impurities, elution was performed to release the precipitated target protein and Ub complexes. 

### 5.12. Western Blot

Protein extraction was performed by adding a mixture of RIPA lysis buffer and 1% PMSF protease inhibitor (Beyotime, Shanghai, China). Then, the cell proteins were collected through centrifugation (10,000× *g* for 30 min at 4 °C). Proteins were separated on 4–20% SAD-PAGE (BeyoGel, Shanghai, China) and transferred onto polyvinylidene difluoride (PVDF) membranes (Millipore, Burlington, VT, USA). A solution of 5% BSA in TBST was used to block the proteins. Then, the clipped membranes were incubated with primary antibodies against TRIM33 (#ab300146, Abcam, Shanghai, China), DHX33 (#ab72451, Abcam, Shanghai, China), SMAD4 (#ab230815, Abcam, Shanghai, China), CTNNB1 (#K002207P, Solarbio, Beijing, China), Ub (#ab134953, Abcam, Shanghai, China), and GAPDH (#60004-1-lg, Proteintech, Wuhan, China) overnight at 4 °C. Secondary antibody conjugated to horseradish peroxidase (HRP) (#7074P2, Cell Signaling, Shanghai, China) was used to treat membranes for 1 h at room temperature. Finally, proteins were visualized by touch chemiluminescence imager (e-BLOT, Shanghai, China).

## Figures and Tables

**Table 1:** Overview of the cancer cohorts.

Cancer Type	PDC Study Name	PDC Study ID	Tumor Sample Size	Normal Sample Size	Paired or Not	Ubiquitinome
Lung Squamous Cell Carcinoma (LSCC)	CPTAC LSCC Discovery Study	PDC000234	108	99	Paired	√
Pancreatic Ductal Adenocarcinoma (PDAC)	CPTAC PDA Discovery Study	PDC000270	141	77	Paired	✕
Glioblastoma (GBM)	CPTAC GBM Discovery Study	PDC000204	99	10	Unpaired	✕
Ovarian Serous Cystadenocarcinoma (OV)	Prospective Ovarian JHU Proteome	PDC000110	83	23	Unpaired	✕
Lung Adenocarcinoma (LUAD)	CPTAC LUAD Discovery Study	PDC000153	110	101	Paired	✕
Head and Neck Squamous Cell Carcinoma (HNSCC)	CPTAC HNSCC Discovery Study	PDC000221	108	67	Paired	✕
Uterine Corpus Endometrial Carcinoma (UCEC)	CPTAC UCEC Discovery Study	PDC000125	104	49	Paired	✕
Breast Invasive Carcinoma (BRCA)	Prospective Breast BI Proteome	PDC000120	96	14	Paired	✕
Clear Cell Renal Cell Carcinoma (CCRCC)	CPTAC CCRCC Discovery Study	PDC000127	110	83	Paired	✕
Hepatocellular carcinoma (HCC)	HBV-Related Hepatocellular Carcinoma	PDC000198	159	159	Paired	✕
Colon Adenocarcinoma (COAD)	Prospective Colon VU Proteome	PDC000109	104	100	Paired	✕
Early Onset Gastric Cancer (EOGC)	Proteogenomics of Gastric Cancer	PDC000214	80	80	Paired	✕

## Data Availability

The CPTAC proteomic data can be openly accessed from the NCI Cancer Research Data Commons repository: https://proteomic.datacommons.cancer.gov/pdc/ (accessed on 12 November 2021). The processed proteomic datasets of the 12 cancer cohorts are available at https://github.com/ZhongyanLI0108/ProteomicsCancers (accessed on 29 November 2023). The LSCC transcriptomic data are available at the Genomic Data Commons: https://gdc.cancer.gov/ (accessed on 19 July 2022). The remaining data are available within Supplementary Information.

## Supplementary Materials

The following supporting information can be downloaded at: https://www.mdpi.com/article/10.3390/ijms25147639/s1, References [7,9,17,18,19,20,21,22,23,24,25,27,36,37,54,55,56,57,58,59,60,61,62,63,64,65,66,67,68,69] are cited in the supplementary materials.
