# Supplementary material for: Multi-Omics Characterization of E3 Regulatory Patterns in Different Cancer Types
Source: Int J Mol Sci. 2024 Jul 11;25(14):7639. doi: 10.3390/ijms25147639 (PMC11276688; doi:10.3390/ijms25147639)
Supplement: Supplementary file 1 [file ijms-25-07639-s001.zip › Supplementary Information_revised.pdf]

Contents

Supplementary Figures

Supplementary Tables

Supplementary Materials and Methods

Supplementary References

Supplementary Figures

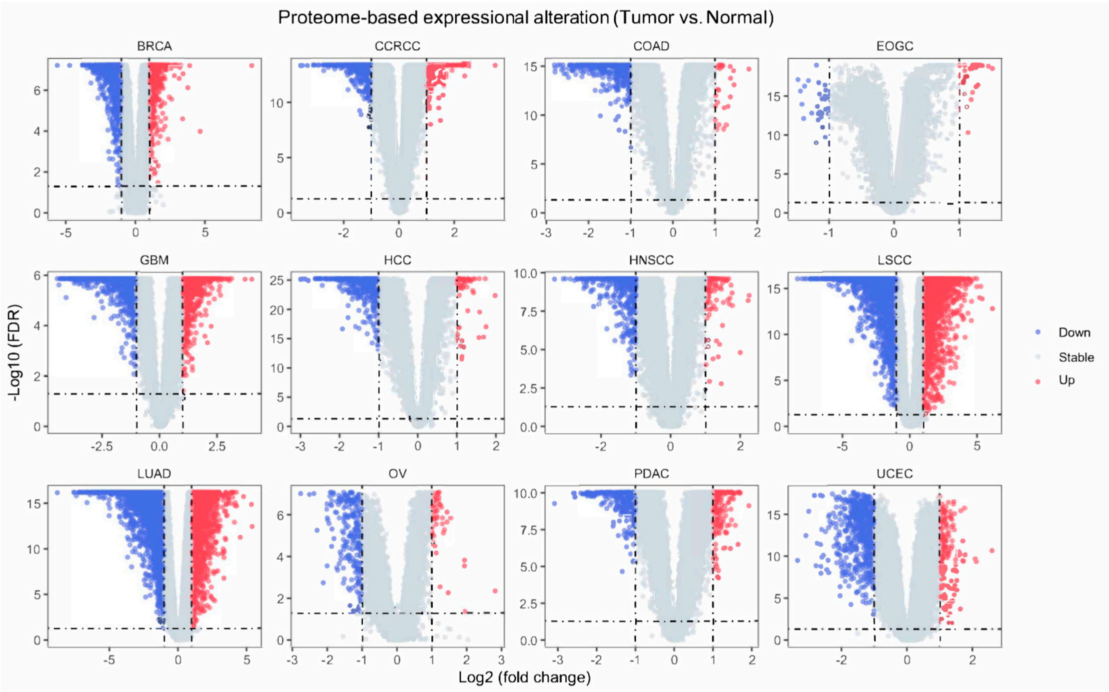

Figure S1. The proteome-based expressional alterations of tumor samples compared with normal samples in 12 cancer types.

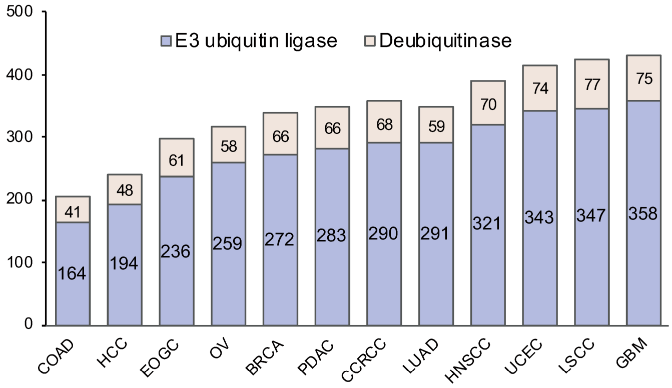

Figure S2. The numbers of E3 ubiquitin ligases and deubiquitinases (DUB) identified from proteomics data sets of 12 types of cancer. The blue bar represents E3, and the gray bar represents DUB.

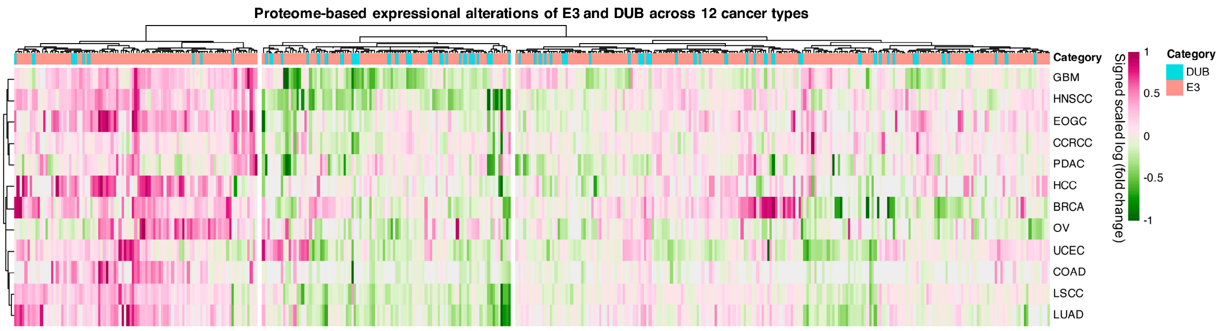

Figure S3. Heatmap of proteome-based expressional alterations combining E3 and DUB (tumor vs. normal)

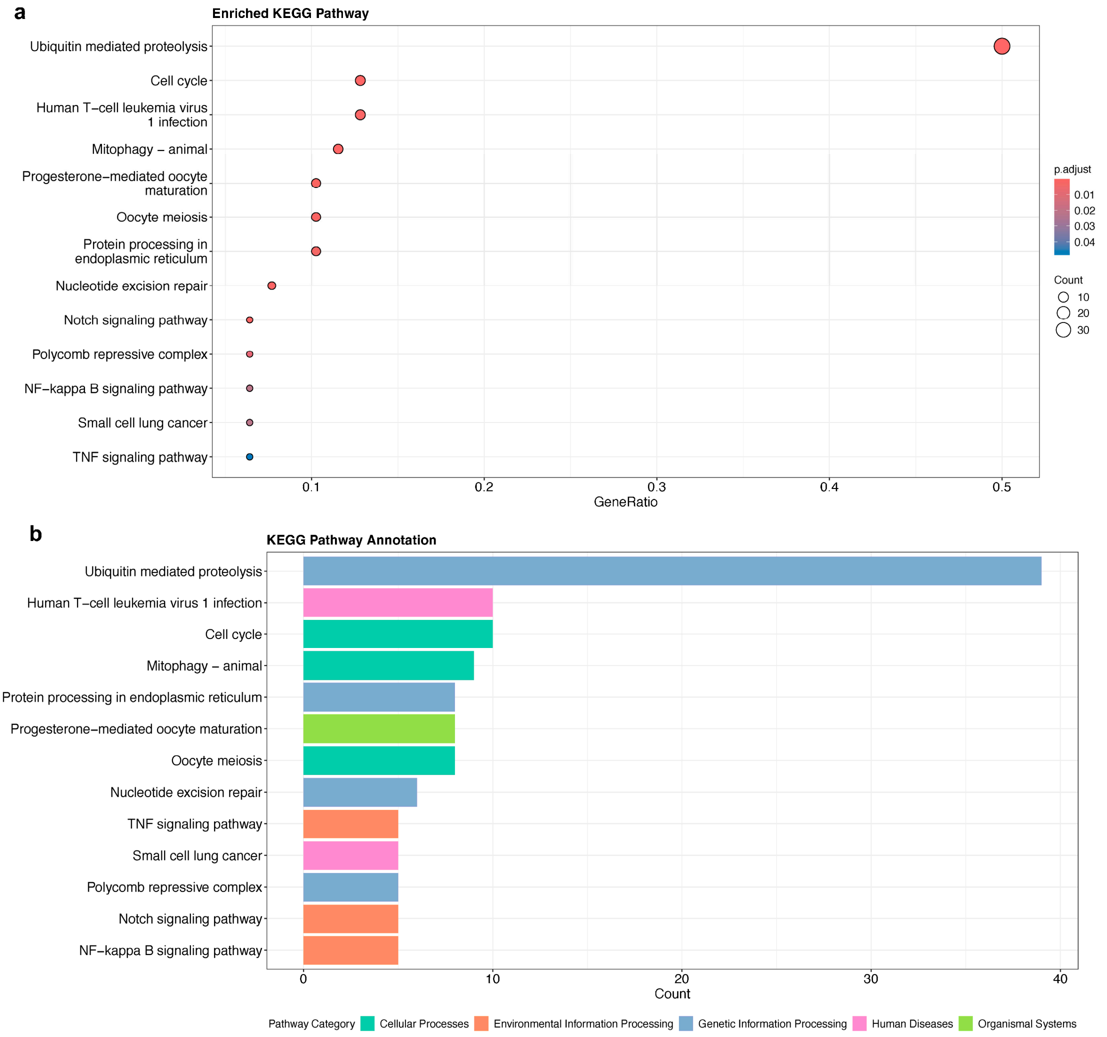

Figure S4. Dot plot of KEGG pathway enrichment analysis for the E3 belonging to the Cluster 3 in Figure 2g and pathway category annotation.

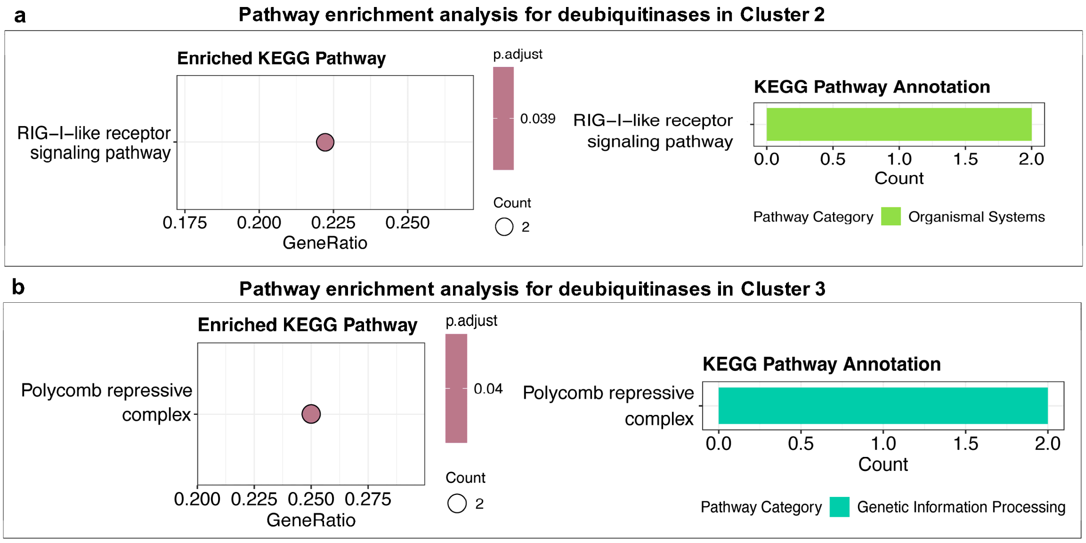

Figure S5. Dot plot of KEGG pathway enrichment analysis for the DUB and pathway category annotation.

a Dot plot of KEGG pathway enrichment analysis for the DUB belonging to the Cluster 2 in **Figure 2j** and pathway category annotation.

b Dot plot of KEGG pathway enrichment analysis for the DUB belonging to the Cluster 3 in **Figure 2j** and pathway category annotation.

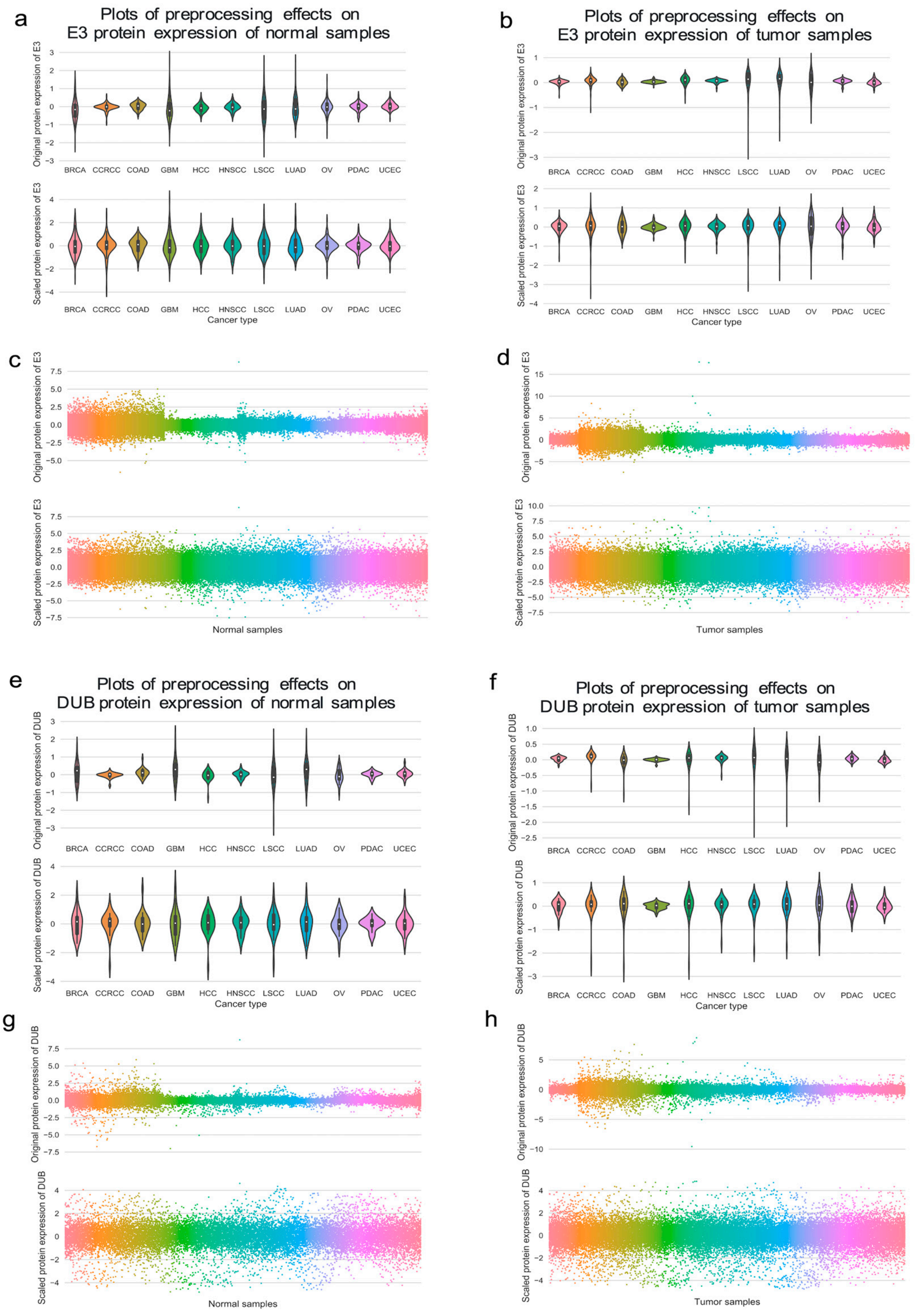

Figure S6. Plots of normalization effects.

a Violin plot of preprocessing effects on E3 protein expression of normal samples.

b Violin plot of preprocessing effects on E3 protein expression of tumor samples.

c Strip plot of preprocessing effects on E3 protein expression of normal samples.

d Strip plot of preprocessing effects on E3 protein expression of tumor samples.

e Violin plot of preprocessing effects on DUB protein expression of normal samples.

f Violin plot of preprocessing effects on DUB protein expression of tumor samples.

g Strip plot of preprocessing effects on DUB protein expression of normal samples.

h Strip plot of preprocessing effects on DUB protein expression of tumor samples.

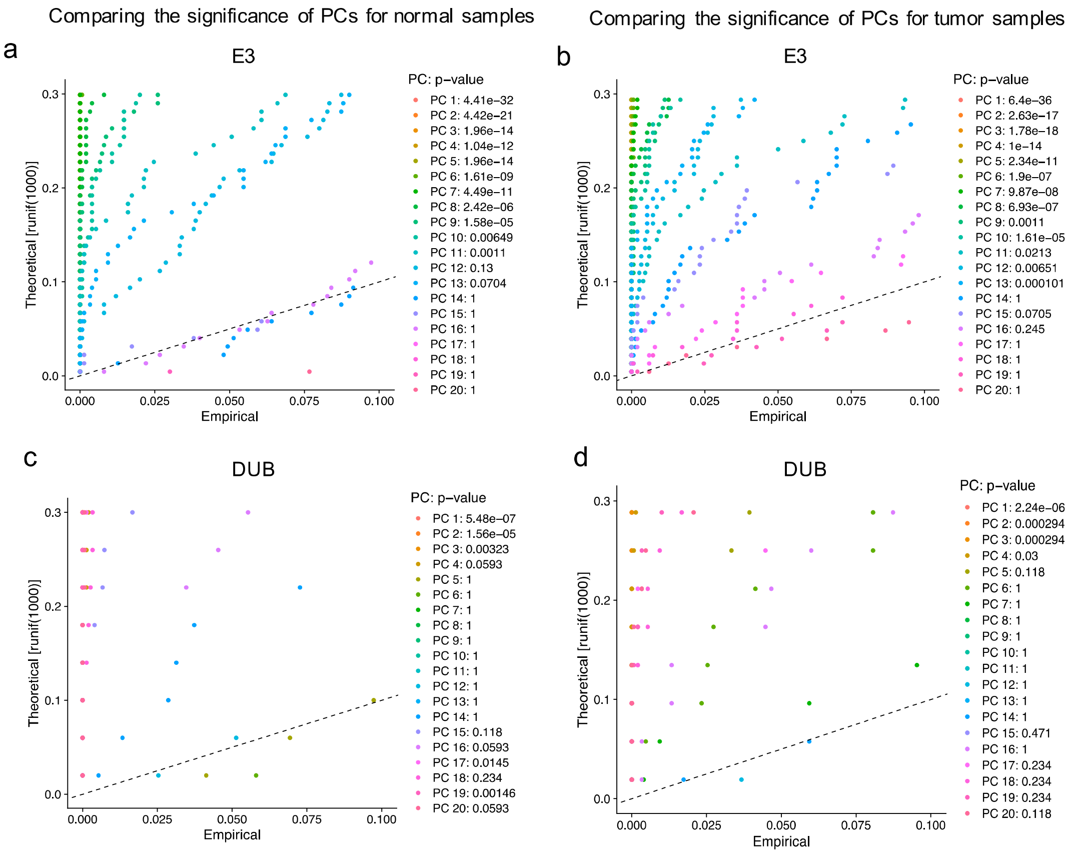

Figure S7. Comparing the significance of PCs when considering E3 or DUB protein expression.

a Comparing the significance of PCs considering E3 for normal samples.

b Comparing the significance of PCs considering E3 for normal samples.

c Comparing the significance of PCs considering DUB for normal samples.

d Comparing the significance of PCs considering DUB for normal samples.

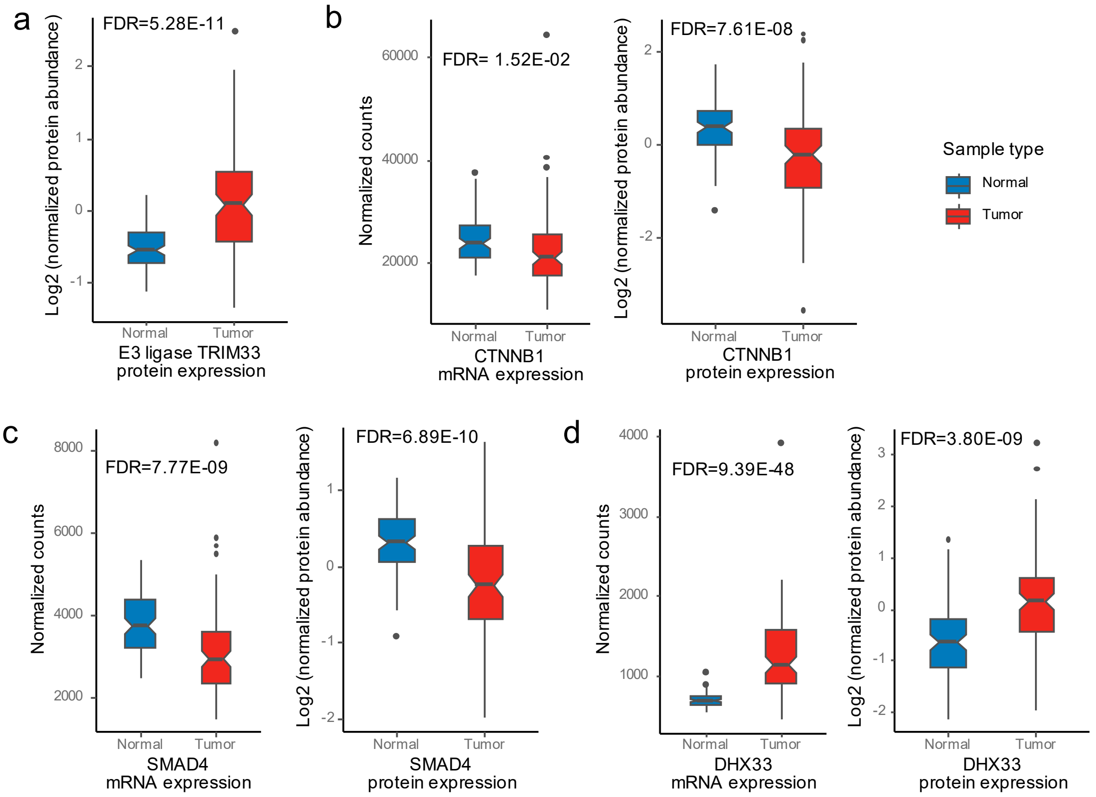

Figure S8. The mRNA and protein expression alterations of TRIM33 and its substrates.

a The TRIM33 protein expression was upregulated significantly.

b The mRNA and protein expression alterations of CTNNB1. Despite only a slight decrease in mRNA expression (FDR > 0.01), there was a significant reduction in protein abundance.

c The mRNA and protein expression alterations of SMAD4.

d The mRNA and protein expression alterations of DHX33.

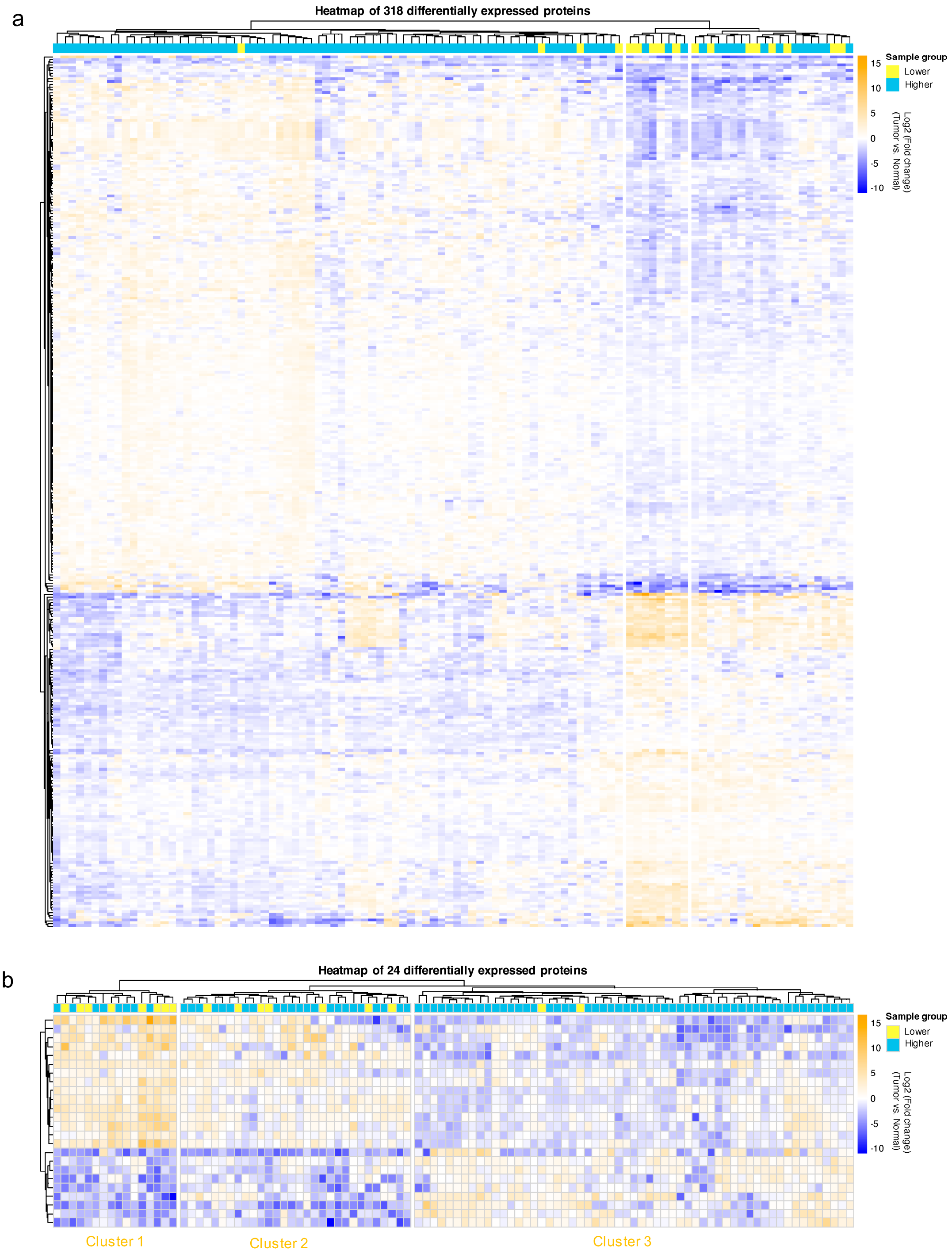

Figure S9. Heatmap of differentially expressed proteins between higher and lower TRIM33 expression groups.

a Heatmap based on 318 differentially expressed proteins.

b Heatmap based on 24 differentially expressed proteins. The higher and lower expression groups have distinct expression patterns for the 24 proteins.

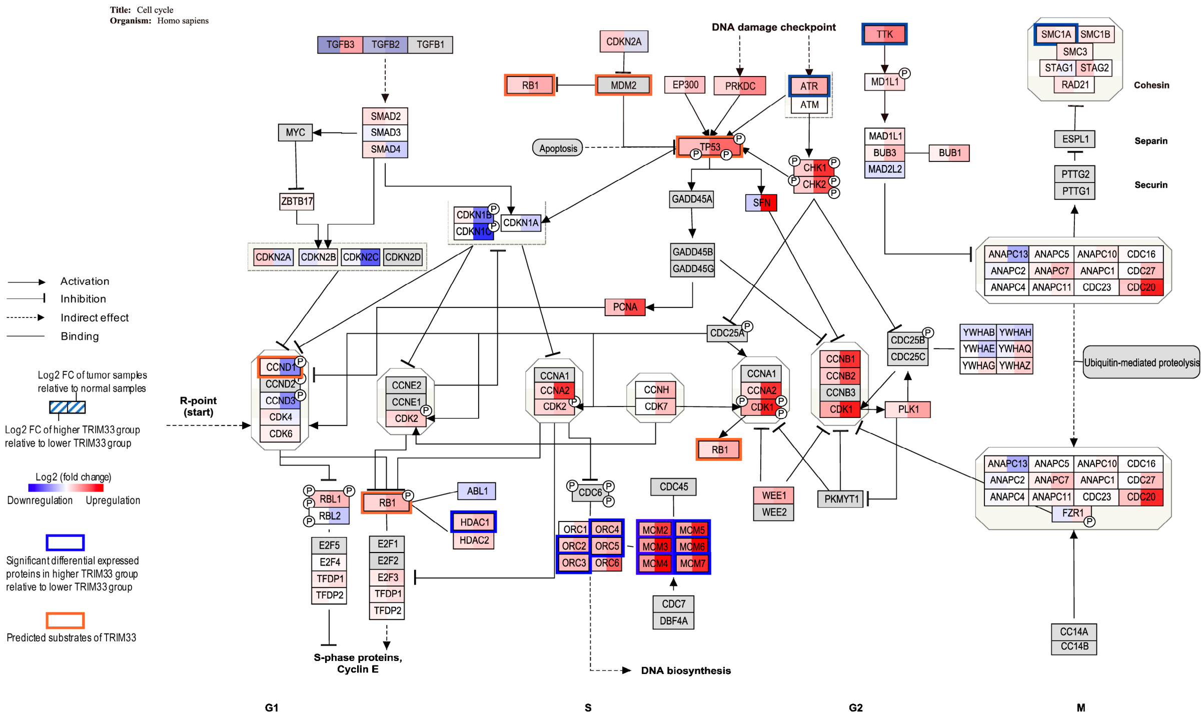

Figure S10. The protein regulatory patterns in the human 'Cell cycle' pathway for the LSCC cohort. Squares in the map represent the proteins in the human cell cycle pathway. Different types of lines and arrows indicate the relations between proteins. The filled color on the left half of the rectangles represents the protein expression alteration between the higher and lower expression groups of TRIM33. The color filled on the right half represents the protein expression change in tumor samples compared to normal samples for that particular protein. Gray rectangles represent the proteins without expression information. Rectangles in blue are significant differential expressed proteins in the higher group relative to the lower group. The predicted substrates of TRIM33 are highlighted in orange.

Supplementary Tables

Table S1[*]. Human E3 ubiquitin ligases.

Table S2[*]. Human deubiquitinases.

Table S3[*]. E3 list of clusters in Figure 2.

Table S4[*]. DUB list of clusters in Figure 2.

Table S5[*]. KEGG pathway enrichment analysis for E3 clusters in Figure 2.

Table S6[*]. KEGG pathway enrichment analysis for DUB clusters in Figure 2.

Table S7[*]. Human E3-substrate interactions.

Table S8. The results of the Kolmogorov-Smirnov test on the correlation between E3 and corresponding substrates in normal and tumor samples.

Table S9[*]. Substrates with up-regulated ubiquitination sites and their up-regulated E3.

Table S10[*]. Up-regulated E3 and their substrates with degradative ubiquitination.

Table S11[*]. Results of survival analysis.

Table S12[*]. Predicted substrates of TRIM33.

* The supplementary tables were presented separately in Excel files.

Table S8. The results of the Kolmogorov-Smirnov test on the correlation between E3 and corresponding substrates in normal and tumor samples.

Cancer type	Statistic	P-value	FDR
GBM	0.17	5.25E-16	5.78E-15
LSCC	0.11	2.31E-09	6.35E-09
LUAD	0.04	3.28E-01	3.28E-01
HCC	0.09	3.23E-02	3.55E-02
OV	0.10	9.07E-04	1.26E-03
HNSCC	0.11	3.69E-07	8.12E-07
UCEC	0.18	5.66E-15	2.08E-14
CCRCC	0.09	9.14E-04	1.26E-03
COAD	0.12	1.65E-03	2.01E-03
PDAC	0.10	4.32E-05	7.91E-05
BRCA	0.23	1.11E-15	6.11E-15

Supplementary Materials and Methods

The Collection of Proteomic Data Sets

We systematically scanned the cohorts in the National Cancer Institute's Proteomic Data Commons (PDC) (1). To ensure the reliability of downstream analyses, the cohorts with less than ten normal or tumor samples were removed. For the cancer types having more than one cohort, only one cohort was chosen. Then, twelve cancer cohorts were retained. Among these, there are ten cancer cohorts from Clinical Proteomic Tumor Analysis Consortium (CPTAC) program, including Lung Squamous Cell Carcinoma (LSCC) (2), Pancreatic Ductal Adenocarcinoma (PDAC) (3), Glioblastoma (GBM) (4), Ovarian Serous Cystadenocarcinoma (OV) (5), Lung Adenocarcinoma (LUAD) (6), Head and Neck Squamous Cell Carcinoma (HNSCC) (7), Uterine Corpus Endometrial Carcinoma (UCEC) (8), Breast Invasive Carcinoma (BRCA) (9), Clear Cell Renal Cell Carcinoma (CCRCC) (10), and Colon Adenocarcinoma (COAD) (11). The other two cancer cohorts, Hepatitis B virus (HBV)-related Hepatocellular Carcinoma (HCC) (12) and Early Onset Gastric Cancer (EOGC) (13), are from the International Cancer Proteogenome Consortium. Tumor samples and paired normal adjacent tissue (NAT) were collected in ten cancer cohorts, while the normal samples of OV and GBM cohorts are not paired with tumor samples (Table 1). Ubiquitinome was only examined for Lung Squamous Cell Carcinoma (LSCC) cohorts. The preprocessed proteomics data sets of eight cohorts were downloaded from supplementary materials of the

corresponding studies, which are cohorts of LSCC, GBM, LUAD, HNSCC, UCEC, CCRCC, HCC, and COAD. The detailed preprocessing methods can be found in the publications of these studies. The other four proteomics data sets of PDAC, OV, BRCA, and EOGC were directly downloaded from the PDC 'Protein Assembly' section.

The Curation of the Protein Sets of Human E3 Ubiquitin Ligases and Deubiquitinases

We obtained the set of E3 ubiquitin ligases by combining the human E3 collected in our previously published UbiNet 2.0 database (Table S1). For the DUB set, we searched UniProt (14) and HUGO Gene Nomenclature Committee (HGNC) (15) against the following keywords: DUBs, DUB, Deubiquitinase, Deubiquitinating enzymes, and ubiquitin-specific protease, which was followed by manually scanning the retrieved proteins. The additional DUB set proposed by Liang's lab (16) was also combined (Table S2).

The Collection of Human E3-substrate Interactions

To dissect the proteome-based relationship between the E3 and their substrates, we curated the human E3-substrate interactions (ESI) from multiple sources. The human ESI retrieved from UbiBrowser 2.0 (17) and UbiNet 2.0 (18) were combined. The additional ESI obtained by manual literature browsing, which are MDM2-ACE2 (19), NEDD4L-CTNNB1 (20), NEDD4L-UBE2T (21), VHL-YAP1 (22), NEDD4L-NOTCH2 (23), HUWE1-TIAM1 (24), SYVN1-SIRT2

(25), RNF115-APC (26). We collected a total of 2922 experimentally validated human ESI (Table S7).

TRIM33-involved Pathway Analysis

The continuous protein expression levels of 49 E3 were used for the Cox proportional hazards model. The upregulation of TRIM33 was identified as the favorable prognostic factor. Then, we systematically investigated the pathways affected by TRIM33. We downloaded 352 human pathways from the KEGG PATHWAY Database (27). Among them, the pathways of the 'Information processing in viruses' category and those belonging to 'Human diseases' but excluding 'Cancer: overview', 'Cancer: specific types', and 'Drug resistance: antineoplastic' categories were removed from the following pathway analysis. The downstream effects of TRIM33 were inferred in two steps. It is known that SMAD4, CTNNB1, and DHX33 are substrates of TRIM33. Firstly, for each substrate, we computed the proteome-based biweight midcorrelations between the substrate and all the other proteins. Biweight midcorrelation is a robust correlation measure that can be calculated by using the 'bicor' function from the WGCNA package (28). Secondly, the proteins were ranked by the absolute values of the correlations, of which the top 500 were used to implement the signaling pathway impact analysis (SPIA). SPIA pathway analysis algorithm offers advantages over traditional pathway enrichment methods by considering pathway topology, protein-protein interactions, and coordinated expression changes. It provides a more accurate assessment of pathway activity and

identifies key regulatory genes, enabling a deeper understanding of complex biological processes (29). Finally, 21 pathways were recognized to be related to CTNNB1, and only the ‘Cell cycle’ pathway was affected by SMAD4. There was no pathway related to DHX33. Then to divide the samples into two groups, the Maximally Selected Rank Statistics were used for the evaluation of a cutpoint model, which was performed by using the ‘maxstat’ package (30). The 318 differentially expressed proteins between the two groups were utilized to conduct the signaling pathway impact analysis.

Supplementary References

1. Thangudu RR, Rudnick PA, Holck M, Singhal D, MacCoss MJ, Edwards NJ, et al. Abstract LB-242: Proteomic Data Commons: A resource for proteogenomic analysis. Cancer Res. 2020;80(16_Supplement):LB-242.
2. Satpathy S, Krug K, Beltran PMJ, Savage SR, Petralia F, Kumar-Sinha C, et al. A proteogenomic portrait of lung squamous cell carcinoma. Cell. 2021;184(16):4348–71.
3. Cao L, Huang C, Zhou DC, Hu Y, Lih TM, Savage SR, et al. Proteogenomic characterization of pancreatic ductal adenocarcinoma. Cell. 2021;184(19):5031–52.
4. Wang LB, Karpova A, Gritsenko MA, Kyle JE, Cao S, Li Y, et al. Proteogenomic and metabolomic characterization of human glioblastoma. Cancer Cell. 2021;39(4):509–28.
5. Hu Y, Pan J, Shah P, Ao M, Thomas SN, Liu Y, et al. Integrated proteomic and

glycoproteomic characterization of human high-grade serous ovarian carcinoma. Cell Rep. 2020;33(3):108276.

6. Gillette MA, Satpathy S, Cao S, Dhanasekaran SM, Vasaikar SV, Krug K, et al. Proteogenomic characterization reveals therapeutic vulnerabilities in lung adenocarcinoma. Cell. 2020;182(1):200–25.
7. Huang C, Chen L, Savage SR, Eguez RV, Dou Y, Li Y, et al. Proteogenomic insights into the biology and treatment of HPV-negative head and neck squamous cell carcinoma. Cancer Cell. 2021;39(3):361–79.
8. Dou Y, Kawaler EA, Zhou DC, Gritsenko MA, Huang C, Blumenberg L, et al. Proteogenomic characterization of endometrial carcinoma. Cell. 2020;180(4):729–48.
9. Krug K, Jaehnig EJ, Satpathy S, Blumenberg L, Karpova A, Anurag M, et al. Proteogenomic landscape of breast cancer tumorigenesis and targeted therapy. Cell. 2020;183(5):1436–56.
10. Clark DJ, Dhanasekaran SM, Petralia F, Pan J, Song X, Hu Y, et al. Integrated proteogenomic characterization of clear cell renal cell carcinoma. Cell. 2019;179(4):964–83.
11. Vasaikar S, Huang C, Wang X, Petyuk VA, Savage SR, Wen B, et al. Proteogenomic analysis of human colon cancer reveals new therapeutic opportunities. Cell. 2019;177(4):1035–49.
12. Gao Q, Zhu H, Dong L, Shi W, Chen R, Song Z, et al. Integrated proteogenomic characterization of HBV-related hepatocellular carcinoma. Cell. 2019;179(2):561–

77.
13. Mun DG, Bhin J, Kim S, Kim H, Jung JH, Jung Y, et al. Proteogenomic characterization of human early-onset gastric cancer. Cancer Cell. 2019;35(1):111–24.
14. Consortium TU. UniProt: the universal protein knowledgebase in 2021. Nucleic Acids Res. 2020 Nov;49(D1):D480–9.
15. Povey S, Lovering R, Bruford E, Wright M, Lush M, Wain H. The HUGO gene nomenclature committee (HGNC). Hum Genet. 2001;109:678–80.
16. Ge Z, Leighton JS, Wang Y, Peng X, Chen Z, Chen H, et al. Integrated genomic analysis of the ubiquitin pathway across cancer types. Cell Rep. 2018;23(1):213–26.
17. Wang X, Li Y, He M, Kong X, Jiang P, Liu X, et al. UbiBrowser 2.0: a comprehensive resource for proteome-wide known and predicted ubiquitin ligase/deubiquitinase–substrate interactions in eukaryotic species. Nucleic Acids Res. 2022;50(D1):D719–28.
18. Li Z, Chen S, Jhong JH, Pang Y, Huang KY, Li S, et al. UbiNet 2.0: a verified, classified, annotated and updated database of E3 ubiquitin ligase–substrate interactions. Database. 2021;2021.
19. Shen H, Zhang J, Wang C, Jain PP, Xiong M, Shi X, et al. MDM2-mediated ubiquitination of angiotensin-converting enzyme 2 contributes to the development of pulmonary arterial hypertension. Circulation. 2020;142(12):1190–204.
20. Zhang W, Zhang R, Zeng Y, Li Y, Chen Y, Zhou J, et al. ALCAP2 inhibits lung

adenocarcinoma cell proliferation, migration and invasion via the ubiquitination of β-catenin by upregulating the E3 ligase NEDD4L. Cell Death Dis. 2021;12(8):755.

21. Chen Y, Hong H, Wang Q, Li J, Zhang W, Chen T, et al. NEDD4L-induced ubiquitination mediating UBE2T degradation inhibits progression of lung adenocarcinoma via PI3K-AKT signaling. Cancer Cell Int. 2021;21(1):1–13.

22. Hu L, Wu H, Jiang T, Kuang M, Liu B, Guo X, et al. pVHL promotes lysosomal degradation of YAP in lung adenocarcinoma. Cell Signal. 2021;83:110002.

23. Lin L, Wu X, Jiang Y, Deng C, Luo X, Han J, et al. Down-regulated NEDD4L facilitates tumor progression through activating Notch signaling in lung adenocarcinoma. PeerJ. 2022;10:e13402.

24. Vaughan L, Tan CT, Chapman A, Nonaka D, Mack NA, Smith D, et al. HUWE1 ubiquitylates and degrades the RAC activator TIAM1 promoting cell-cell adhesion disassembly, migration, and invasion. Cell Rep. 2015;10(1):88–102.

25. Liu L, Yu L, Zeng C, Long H, Duan G, Yin G, et al. E3 ubiquitin ligase HRD1 promotes lung tumorigenesis by promoting sirtuin 2 ubiquitination and degradation. Mol Cell Biol. 2020;40(7):e00257-19.

26. Wu XT, Wang YH, Cai XY, Dong Y, Cui Q, Zhou YN, et al. RNF115 promotes lung adenocarcinoma through Wnt/β-catenin pathway activation by mediating APC ubiquitination. Cancer Metab. 2021;9(1):1–12.

27. Kanehisa M, Furumichi M, Sato Y, Kawashima M, Ishiguro-Watanabe M. KEGG for taxonomy-based analysis of pathways and genomes. Nucleic Acids Res. 2023;51(D1):D587–92.

28. Langfelder P, Horvath S. WGCNA: an R package for weighted correlation network analysis. BMC Bioinformatics. 2008;9(1):1–13.

29. Tarca AL, Draghici S, Khatri P, Hassan SS, Mittal P, Kim J sun, et al. A novel signaling pathway impact analysis. Bioinformatics. 2009;25(1):75–82.

30. Hothorn T, Lausen B. Maximally selected rank statistics in R. R News. 2002;2(1):3–5.
